# Identifying subgroups of childhood obesity by using multiplatform metabotyping

**DOI:** 10.3389/fmolb.2023.1301996

**Published:** 2023-12-20

**Authors:** David Chamoso-Sanchez, Francisco Rabadán Pérez, Jesús Argente, Coral Barbas, Gabriel A. Martos-Moreno, Francisco J. Rupérez

**Affiliations:** ^1^ Centro de Metabolómica y Bioanálisis (CEMBIO), Facultad de Farmacia, Universidad San Pablo-CEU, CEU Universities, Boadilla del Monte, Spain; ^2^ Departamento Economía Aplicada I, Universidad Rey Juan Carlos, Madrid, Spain; ^3^ Department of Pediatrics and Pediatric Endocrinology, Hospital Infantil Universitario Niño Jesús, Instituto de Investigación Sanitaria La Princesa, Universidad Autónoma de Madrid, Madrid, Spain; ^4^ CIBER Fisiopatología de la Obesidad y Nutrición (CIBEROBN), Instituto de Salud Carlos III, Madrid, Spain; ^5^ IMDEA Food Institute, Madrid, Spain

**Keywords:** multiplatform metabolomics, factor analysis, data integration, obesity, childhood, leptin-melanocortin pathway

## Abstract

**Introduction:** Obesity results from an interplay between genetic predisposition and environmental factors such as diet, physical activity, culture, and socioeconomic status. Personalized treatments for obesity would be optimal, thus necessitating the identification of individual characteristics to improve the effectiveness of therapies. For example, genetic impairment of the leptin-melanocortin pathway can result in rare cases of severe early-onset obesity. Metabolomics has the potential to distinguish between a healthy and obese status; however, differentiating subsets of individuals within the obesity spectrum remains challenging. Factor analysis can integrate patient features from diverse sources, allowing an accurate subclassification of individuals.

**Methods:** This study presents a workflow to identify metabotypes, particularly when routine clinical studies fail in patient categorization. 110 children with obesity (BMI > +2 SDS) genotyped for nine genes involved in the leptin-melanocortin pathway (CPE, MC3R, MC4R, MRAP2, NCOA1, PCSK1, POMC, SH2B1, and SIM1) and two glutamate receptor genes (GRM7 and GRIK1) were studied; 55 harboring heterozygous rare sequence variants and 55 with no variants. Anthropometric and routine clinical laboratory data were collected, and serum samples processed for untargeted metabolomic analysis using GC-q-MS and CE-TOF-MS and reversed-phase U(H)PLC-QTOF-MS/MS in positive and negative ionization modes. Following signal processing and multialignment, multivariate and univariate statistical analyses were applied to evaluate the genetic trait association with metabolomics data and clinical and routine laboratory features.

**Results and Discussion:** Neither the presence of a heterozygous rare sequence variant nor clinical/routine laboratory features determined subgroups in the metabolomics data. To identify metabolomic subtypes, we applied Factor Analysis, by constructing a composite matrix from the five analytical platforms. Six factors were discovered and three different metabotypes. Subtle but neat differences in the circulating lipids, as well as in insulin sensitivity could be established, which opens the possibility to personalize the treatment according to the patients categorization into such obesity subtypes. Metabotyping in clinical contexts poses challenges due to the influence of various uncontrolled variables on metabolic phenotypes. However, this strategy reveals the potential to identify subsets of patients with similar clinical diagnoses but different metabolic conditions. This approach underscores the broader applicability of Factor Analysis in metabotyping across diverse clinical scenarios.

## Introduction

Childhood obesity prevalence has increased worldwide in the last decades, including a higher incidence of severe and early onset cases, particularly after the COVID-19 outbreak lockdown (Choi et al., 2023), enhancing the known risk for long-term consequences in these patients (Rupérez et al., 2020; Handakas et al., 2022). Children with obesity are more susceptible to maintain their adiposity in adult life, increasing the risk of multiple comorbidities at an early age, including type 2 diabetes mellitus (T2DM), dyslipidemia, cardiovascular disease (CVD), hypertension, obstructive sleep apnea, cancer and steatohepatitis (da Fonseca et al., 2017; Cote et al., 2013; Butte et al., 2015; Carde et al., 2020; Berger, 2018; Wahl et al., 2012). Obesity has a multifactorial etiology, with lifestyle, including nutritional and physical activity habits, as well as other environmental factors, interacting with an individual’s unique genetic background to determine a person’s risk to develop obesity (Trang and Grant, 2023). Among the large set of genes influencing obesity, those in the leptin-melanocortin satiety signaling pathway are the most determinant known to date, with homozygous mutations in some causing early onset severe obesity with hyperphagia (Jackson et al., 1997; Chiurazzi et al., 2020; Trang and Grant, 2023). The role of heterozygous variants is under investigation (Trang and Grant, 2023), particularly those with confirmed pathogenicity or high Combined Annotation Dependent Depletion (CADD) scores of “deleteriousness” with low population prevalence [heterozygous rare sequence variants (HetRSVs)]. Additionally, variants in glutamate receptors, pivotal in neuron signaling have also been described in patients with severe obesity (Bell et al., 2005; Fuente-Martín et al., 2016; Serra-Juhé et al., 2017; Fairbrother et al., 2018; Chiurazzi et al., 2020).

Whereas some obesity-associated comorbidities commonly identified in adults can also be observed in children with obesity, others such as T2DM are far less common, with insulin resistance (IR) usually found as the first step in carbohydrate metabolism impairment in childhood obesity (Martos-Moreno et al., 2019). Additionally, not every patient with obesity shows the same risk to develop comorbidities, with the “metabolically healthy obesity” designation proposed for those patients with obesity, even severe obesity, but with no metabolic comorbidities (Wan Mohd Zin et al., 2022). However, this term is under discussion and this condition is known to evolve throughout life in relationship to weight control (Martos-Moreno et al., 2021). The term “metabotype” was defined by Gavaghan et al. (Gavaghan et al., 2000) as “a probabilistic multiparametric description of an organism in a given physiological state based on analysis of its cell types, biofluids, or tissues.” Subsequently, this definition has been repeatedly used (Waldram et al., 2009; Sullivan et al., 2011; Palmnäs et al., 2020), establishing itself as the characterization of the metabolic phenotype of an individual. Recent advances in high-throughput sequencing technologies and computational methods have enabled the generation of large and complex *-omics* datasets, providing an unprecedented opportunity to integrate simultaneous information from multiple molecular levels to investigate the complexity of biological systems (T et al., 2019; Park et al., 2022; Argelaguet et al., 2020; Tanabe et al., 2021; Clark et al., 2021). The integration of various *-omics* data, including genomics, transcriptomics, proteomics, metabolomics, and epigenomics, can help to understand the intricate interplay between different biological molecules and pathways, enabling the identification of key regulators and mechanisms of disease (Hoadley et al., 2014; Meng et al., 2016; Marabita et al., 2022). In metabolomics, a multiplatform strategy combines many analytical tools to study the entire metabolic phenotype. Combining data from multiple sources could result in a better comprehension of the underlying biological mechanisms driving complex diseases including cancer, obesity, and cardiovascular disease (Hoadley et al., 2014; Meng et al., 2016; Marabita et al., 2022; Park et al., 2022). Although much effort has been made in recent years to integrate information from different *-omics* technologies into a single analysis, it is still usual to use a multiplatform strategy individually (T et al., 2019; Argelaguet et al., 2020; Tanabe et al., 2021; Zhang et al., 2022).

Factor Analysis is a multivariate statistical technique that can identify underlying patterns in a large dataset by reducing the number of variables into a smaller number of factors (Lee et al., 2019; Acal et al., 2020). In the context of metabolomics, Factor Analysis can identify metabolite modules, which are groups of metabolites that are highly correlated and potentially involved in a common biological process. This approach provides a more comprehensive understanding of the underlying molecular mechanisms of disease and can identify potential biomarkers and therapeutic targets that may not be identifiable using individual metabolites. Recent studies have demonstrated the potential of Factor Analysis in metabolomics for identifying metabolite modules in various fields of research including cancer biology, metabolic disorders, and neurodegenerative diseases (Shen et al., 2009; Zhao et al., 2013; Argelaguet et al., 2018; Kamleh et al., 2018; Clark et al., 2021). However, there are several challenges associated with the application of Factor Analysis in metabolomics. One of the key challenges is the selection of an appropriate Factor Analysis method (principal component analysis, common Factor Analysis, maximum likelihood method, etc.) which depends on the specific research questions and the characteristics of the metabolomics dataset. Also, multicollinearity is a serious problem that must be solved before performing a Factor Analysis (Chan et al., 2022). Another challenge is the interpretation of the identified metabolite modules, as it may be difficult to determine the biological relevance of the modules. This challenge can be addressed by integrating the results of Factor Analysis with other omics data types, such as genomics, transcriptomics, and proteomics, to provide a more comprehensive understanding of the underlying biological processes. Combining Factor Analysis with a hierarchical clustering analysis enables one to classify patients considering all metabolic features detected by a multi-platform approach; to identify patient subgroups based on their metabotype and to provide the optimal treatment for each patient rather than based upon the usual anthropometric and routine laboratory parameters used in the clinical setting and even over the presence or absence of HetRSVs in relevant genes in the studied pathology. Such strategy becomes even more powerful when there is no classification available, or the main goal of the research is to unveil the minimum set of parameters which allow for classification/stratification.

## Patients, materials and methods

**FIGURE 1:**
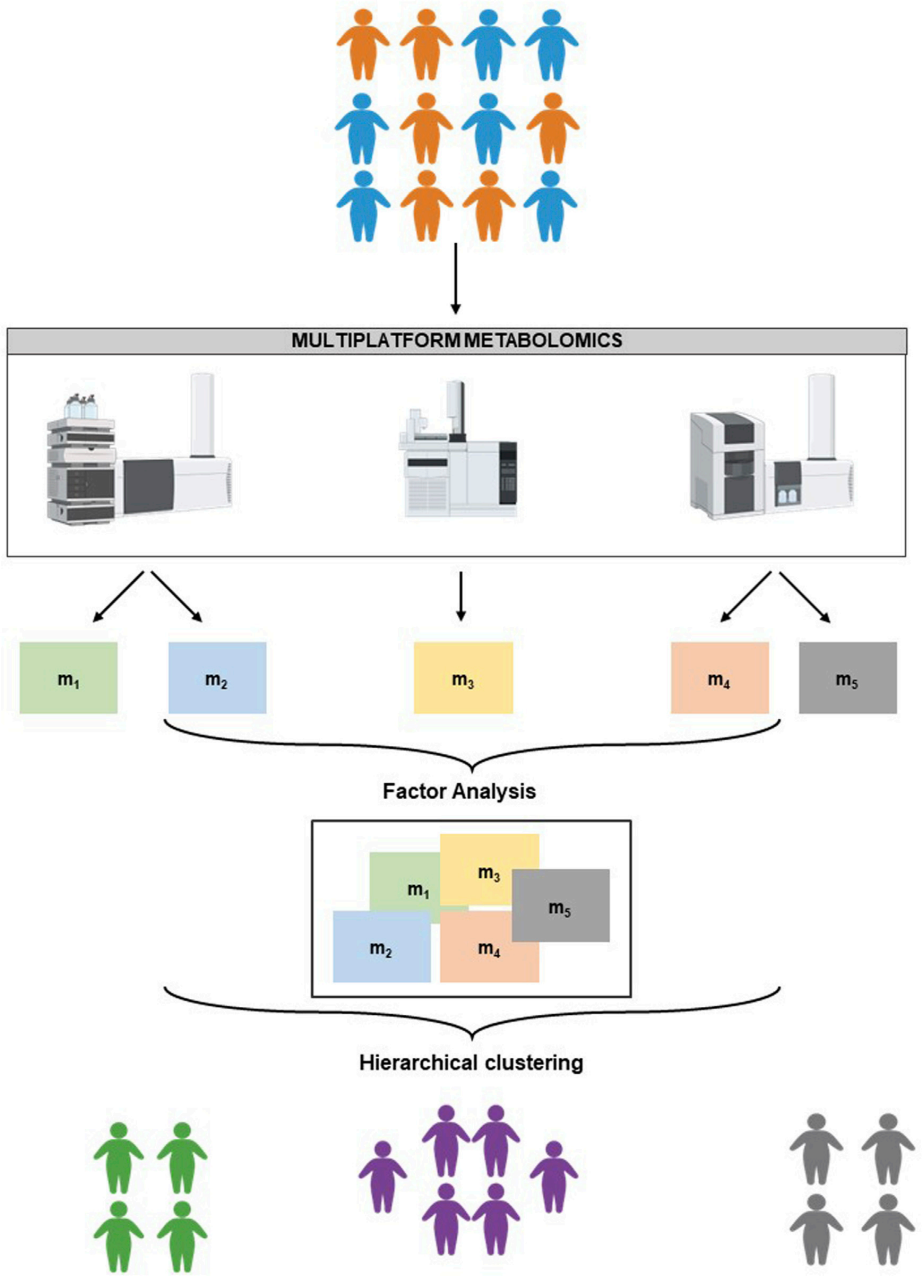
Schematic representation of the experimental design.

We tested a multi-platform strategy in combination with Factor Analysis and hierarchical clustering for personalized approaches in the treatment of obesity (Figure 1).

### Patients

One hundred and ten children and adolescents (57 females/53 males) affected with severe obesity referred to a specialized clinic in a third level monographic pediatric hospital and genotyped for nine genes in the leptin-melanocortin pathway downstream of the leptin receptor, and two glutamate receptor genes (Table 1) were studied: 55 of them harboring one heterozygous rare sequence variant [HetRSV, defined as populational frequency <0.01 and with a Combined Annotation Dependent Depletion (CADD) score of “deleteriousness” > 20] and/or confirmed pathogenicity according to ACMG criteria in the studied genes [*CPE* (*n* = 5), *MC3R* (*n* = 5), *MC4R* (*n* = 5), *MRAP2* (*n* = 5), *NCOA1* (*n* = 7), *PCSK1* (*n* = 5), *POMC* (*n* = 5), *SH2B1* (*n* = 5), *SIM1* (*n* = 5), *GRM7* (*n* = 4) or *GRIK1* (*n* = 4)] and 55 with no detected variants.

**TABLE 1:** Gene list.

*CPE* (MIM* 114855. Carboxypeptidase E)
*GRIK1* (MIM* 138245. GLUTAMATE RECEPTOR, IONOTROPIC, KAINATE 1)
*GRM7* (MIM* 604101. GLUTAMATE RECEPTOR, METABOTROPIC, 7)
*MC3R* (MIM* 155540. MELANOCORTIN 3 RECEPTOR)
*MC4R* (MIM* 155541. MELANOCORTIN 4 RECEPTOR)
*MRAP2* (MIM* 615410. MELANOCORTIN 2 RECEPTOR ACCESSORY PROTEIN 2)
*NCOA1* (MIM* 602691. NUCLEAR RECEPTOR COACTIVATOR 1) (Alternative nomenclature: SRC1)
*PCSK1* (MIM* 162150. PROPROTEIN CONVERTASE, SUBTILISIN/KEXIN-TYPE, 1)
*POMC* (MIM* 176830. PROOPIOMELANOCORTIN)
*SH2B1* (MIM* 608937. SH2B ADAPTOR PROTEIN 1)
*SIM1* (MIM* 603128. SIM bHLH TRANSCRIPTION FACTOR 1)

The whole cohort mean age and standardized body mass index (BMI) were 11.01 ± 3.36 years and 4.20 ± 2.20 SDS, respectively with no differences between groups (with vs. without variants) in age, BMI-SDS, routine laboratory metabolic and hormonal features nor in sex, ethnicity, or pubertal status distribution. Their main anthropometric and metabolic features are summarized and compared in Table 2.

**TABLE 2:** Anthropometric and metabolic features.

Clinical parameters	Whole cohort	Variant carriers	NO variant	Variant carriers vs. NO
Age (years)	11.01 ± 3.36	11.12 ± 3.47	10.91 ± 3.28	N.S.
Height (SDS)	0.84 ± 1.12	0.70 ± 1.05	0.99 ± 1.19	N.S.
BMI-SDS	4.20 ± 2.20	4.29 ± 2.42	4.11 ± 1.99	N.S.
Fasting glucose (mg/dL)	91.10 ± 6.81	90.99 ± 5.98	91.91 ± 7.51	N.S.
Glucose at 120′in OGTT (mg/dL)	120.82 ± 18.87	118.11 ± 13.31	123.31 ± 18.29	N.S.
Fasting insulin (µU/mL)	15.13 ± 7.28	14.83 ± 7.56	15.42 ± 7.05	N.S.
HOMA index	3.45 ± 1.75	3.34 ± 1.78	3.56 ± 1.72	N.S.
WBISI index	3.23 ± 1.67	3.35 ± 1.63	3.13 ± 1.71	N.S.
HbA1c (%)	5.43 ± 0.30	5.44 ± 0.24	5.41 ± 0.35	N.S.
Uric acid (mg/dL)	5.18 ± 1.16	5.04 ± 1.21	5.32 ± 1.11	N.S.
GOT (U/L)	27.72 ± 7.35	26.80 ± 6.73	28.68 ± 7.89	N.S.
GPT (U/L)	22.22 ± 9.27	21.38 ± 8.48	23.19 ± 10.03	N.S.
GGT (U/L)	14.16 ± 4.72	13.91 ± 4.31	14.42 ± 5.13	N.S.
HDL-c (mg/dL)	46.72 ± 14.02	46.63 ± 15.93	46.82 ± 11.90	N.S.
LDL-c (mg/dL)	96.21 ± 27.23	96.82 ± 30.57	97.62 ± 23.55	N.S.
Triglycerides (mg/dL)	78.69 ± 48.61	75.58 ± 42.40	81.85 ± 54.43	N.S.
Free thyroxine (T4) (ng/dL)	0.94 ± 0.13	0.95 ± 1.09	0.92 ± 01.54	N.S.
TSH (µU/mL)	2.78 ± 1.57	2.91 ± 1.64	2.65 ± 1.51	N.S.
IGF-I (ng/mL)	310.8 ± 171.7	324.67 ± 191.76	297.77 ± 151.24	N.S.
IGFBP-3 (µg/mL)	4.90 ± 0.99	4.93 ± 0.97	4.86 ± 1.02	N.S.
25-OH-Vitamin D (ng/mL)	23.36 ± 9.22	23.55 ± 8.69	23.21 ± 9.74	N.S.
Intact PTH (pg/mL)	57.13 ± 21.92	57.03 ± 22.04	57.21 ± 22.13	N.S.

Abbreviations: BMI-SDS, Standardized body mass index (Z-score); OGTT, oral glucose tolerance test; HOMA, homeostatic model assessment; WBISI, Whole-body insulin sensitivity index; HDL-c, High density lipoprotein cholesterol; LDL-c, Low density lipoprotein cholesterol; GGT, Gamma-glutamyltransferase; GOT, Glutamic-oxalacetic transaminase; GPT, glutamic-pyruvic transaminase Alanine aminotransferase; IGF-1, Insulin-like growth factor 1; IGFBP3, Insulin-like Growth Factor-binding Protein 3; HbA1c, hemoglobin A1c; TSH, thyroid-stimulating hormone; PTH, parathyroid hormone.

All patients and their parents or guardians gave informed written consent as required by the ethics committee at the University Hospital Niño Jesús, which had previously approved the study in accordance with the “Ethical Principles for Medical Research Involving Human Subjects” adopted in the Declaration of Helsinki by the World Medical Association (64th WMA General Assembly, Fortaleza, Brazil, October 2013).

### Methods

Weight, height, BMI, waist circumference, and systolic and diastolic blood pressure (BP, mean of three measurements) were recorded and standardized (Cole et al., 2000; Ferná et al., 2004) in all patients. A 12‐hour fasting serum sample (drawn, immediately processed, aliquoted and stored at −80°C until assayed) was used to determine glucose, insulin, HbA1c, lipid profile, uric acid, GOT, GPT, GGT, free thyroxin, thyroid stimulating hormone, IGF-I, IGFBP-3, 25-OH-vitamin D and intact parathyroid hormone (iPTH) levels by standardized assays as previously reported (Martos-Moreno et al., 2019). An oral glucose tolerance test (OGTT, 1.75 g/kg, maximum 75 g) for glucose and insulin determination at 30, 60 and 120 min was performed, HOMA (homeostatic model for insulin resistance) and WBISI (whole body insulin sensitivity) indexes were calculated as previously reported (Martos-Moreno et al., 2019).

### Multiplatform untargeted metabolomics analysis

#### Sample treatment

Serum metabolite extraction was carried out according to previously reported standard protocols (Garcia and Barbas, 2011; Pellegrino et al., 2014; Naz et al., 2015). Briefly, for LC-MS analysis, 40 µL of serum was mixed with 800 µL of a cold mixture (−20°C) of methanol:MTBE:Chloroform (1.33:1:1, v/v/v) with Sphinganine (D17:0) and palmitic acid-d31 as internal standards. Samples were vortexed for 30 s and shaken for 20 min at maximum speed at room temperature. Next, samples were centrifuged (13,200 rpm, room temperature, 5 min). After centrifugation, supernatant was directly injected into the system. For GC-MS analysis, protein precipitation was achieved by mixing one volume of serum with three volumes of cold (−20°C) acetonitrile with 25 ppm of palmitic acid-d31 as internal standard, followed by methoximation with O-methoxyamine hydrochloride (15 mg/mL) in pyridine, and sylation with BSTFA: TMCS (99:1). Finally, 20 ppm of tricosane in heptane was added as second internal standard. For CE-MS analysis, 100 µL of serum was mixed with 100 µL of 0.2 M formic acid containing 5% acetonitrile and 0.4 mM methionine sulfone, 2 mM paracetamol and 0.5 mM 4-Morpholineethanesulfonic acid, 2-(N-Morpholino) ethanesulfonic acid (MES) as internal standards. The sample was transferred to an ultracentrifugation device (Millipore Ireland Ltd., Carrigtohill, Ireland) with a 30 kDa protein cutoff for deproteinization through centrifugation (2000 × *g*, 4°C, 90 min). Detailed version of the sample treatment protocols, the reagents, solvents, standards used for the sample treatment and subsequent analyses, and the analytical setup for the LC–MS, GC–MS, and CE–MS analysis are described in Supplementary Material. Quality control samples (QC) were prepared by pooling and mixing equal volumes of each serum sample and treated as independent samples to check the performance of the systems and the reproducibility of the sample treatment. Then, samples were randomized, and QCs were injected at the beginning, along the sequence, and at the end of the batch. Finally, two blank solutions were prepared along with the rest of the samples and analyzed at the beginning and at the end of the analytical sequence (Dudzik et al., 2018).

### LC-MS and CE-MS data pre-processing

The raw data obtained after the LC-MS and CE-MS analysis were processed using Agilent Technologies MassHunter Profinder B.10.0.2.162 (Santa Clara, United States) to clean the background noise and unrelated ions. This algorithm aligns all ions across the samples using mass and retention time (RT) to create a single spectrum for each group of compounds, and finally obtaining a structured data matrix and appropriate format. Missing values were imputed using the k-nearest neighbors (kNN) algorithm (Armitage et al., 2015) in Matlab R2022a software (Mathwoks, Inc., Natick, United States). Then, the data matrix was filtered by coefficient of variation (CV), maintaining those signals that, in the QCs, presented a CV below 30%. The filtered data matrix was imported into SIMCA 17 Sartorius (Goettingen, Germany) to generate a PCA and thus observe the trend of the QCs, detect possible outliers, and look for natural and analytical trends of the samples. To reduce the impact of instrumental and experimental variations that can interfere with the ability to detect biological variations, a correction method called “quality control samples and support vector regression (QC-SVRC)” was used to adjust the data (Kuligowski et al., 2015) implemented in MATLAB R2022a and then normalized by internal standard (IS).

### Data pre-processing and compound identification GC–MS analysis

The chromatograms obtained from each of the serum samples, the QCs, and the IS signal were visually examined to ensure the quality of the obtained profiles and the reproducibility of the IS signal using Agilent MassHunter Qualitative B.10.0.010305.0 software (Santa Clara, United States). Deconvolution and metabolite identification was achieved using the Agilent MassHunter Unknowns Analysis Tool 10.0 (Santa Clara, United States). The software assigned a chemical identity to each of the signals obtained after the search in two commercial libraries: the Fiehn library version 2013, and the NIST library version 2017 and “in-house” libraries. The identities were assigned according to the retention time (RT) and spectra extracted during deconvolution when the software compared them with each compound included in the libraries. Next, the obtained data were aligned using the MassProfiler Professional B.15.1 software (Agilent Technologies) (Santa Clara, United States) and exported to Agilent MassHunter Quantitative Analysis version B10.0.707.0 (Santa Clara, United States) to assign the main ions and the integration of each of the signals. As in the LC-MS and CE-MS analysis, the missing values were estimated using the kNN (k-nearest neighbors) algorithm (Armitage et al., 2015). Experimental and analytical variations were excluded by performing normalization. As in the LC-MS and CE-MS analysis the data matrix was normalized by applying the QC-SRVC correction, normalized by internal standard, and filtered by CV in the QCs (Kuligowski et al., 2015).

### Compound identification LC-MS and CE-MS analysis

For the metabolite tentative annotation initially the m/z was searched against multiple databases available online, including METLIN (http://metlin.scripps.edu), LipidsMAPS (http://lipidMAPS.org) and KEGG (http://www.genome.jp/kegg/), all of which have been joined into an “in-house” developed search engine, CEU MassMediator (http://ceumass.eps.uspceu.es/) (Gil-de-la-Fuente et al., 2019). Aiming to obtain additional information for some identities, HMDB (http://hmdb.ca) was also consulted. In parallel, three complementary software, MS-DIAL (http://prime.psc.riken.jp/), LipidAnnotator (Agilent Technologies) and LipidHunter (Ni et al., 2017; Koelmel et al., 2020; Tsugawa et al., 2020) by fragmentation mass/mass spectra were used for LC-MS identification. Features that were tentatively assigned to metabolites from the databases were based on (1): mass accuracy (maximum error mass 20 ppm) (2), isotopic pattern distribution (3), possibility of cation and anion formation (4), adduct formation (5), elution order of the compounds based on the chromatographic conditions, and (6) MS/MS spectra. Additionally, an “in-house” CE-MS library built with authentic standards was used to compare the relative migration time (RMT) to increase the confidence of the annotations. The confidence levels established by the Compound Identification group of the Metabolomics Society at the 2017 annual meeting of the Metabolomics Society (Brisbane, Australia) have been used. The new identification levels (Blaženović et al., 2018) range from level 0 with full identification based on knowledge of its 3D structure, level 1 2D confidence using comparison with reference standards, level 2 probable structure when compared with database, level 3 possible structure or class and level 4 as unidentified compound.

### Statistical analysis

Statistical analysis was carried out by univariate (UVA, Matlab R2022a) and multivariate analysis [MVA, SIMCA 17, R v4.1.2 and IBM SPSS v27 (Armonk, NY, United States)]. For the UVA, parametric (unpaired *t*-test) with a Benjamini–Hochberg False Discovery Rate *post hoc* correction (q < 0.05) was applied. For MVA, the PCA plot, PLS-DA plot and OPLS-DA plot was built. The data matrix was analyzed using unsupervised machine learning using R environment (https://www.r-project.org/), applying clustering technique to obtain pattern in our data independently of the initial groups.

The raw data from the various analytical platforms were merged using Factor Analysis and hierarchical clustering to generate a broad perspective of the results and to assign metabotypes based on the metabolic phenotypes of each patient with obesity. The whole process of Factor Analysis and hierarchical clustering was carried out by using IBM SPSS software and Microsoft Excel. First, the Pearson correlations between the variables in each of the matrices were analyzed to eliminate multicollinearity. Correlations between the various matrices (inter-matrix correlations) were examined after filtering by the specific correlations of each matrix (intra-matrix correlations). The individual matrices with the resulting variables were subjected to principal component analysis with varimax rotation (Acal et al., 2020) to reduce dimensionality. Three rules were applied to select the number of principal components in each of the individual matrices, the “Scree plot elbow,” the Kaiser-Guttman test (Eigenvalue greater than unity) and a total explained variance greater than 60% (Cattell, 1966). The principal component scores have been analyzed. The variables that present a principal component score higher than 0.5 in any of the selected components and that do not present double saturation are kept for the subsequent Factor Analysis. We consider double saturation to be when the smallest difference in the principal component score of a variable between two components is less than 0.1. The resulting variables have been subjected to a Factor Analysis by maximum likelihood (Babakus et al., 1987) with varimax rotation of each of the matrices separately to further reduce dimensionality. The same three rules were applied as in the PCA. For the final Factor Analysis, variables that displayed double saturation or had factor scores lower than 0.5 in any of the chosen factors were excluded. Finally, all the resulting variables were pooled into a single matrix after applying all these filters and a Factor Analysis was performed using a maximum likelihood extraction method and a varimax rotation method. All variables that entered the combined Factor Analysis of the different platforms were identified using internal databases and mass/mass fragmentation spectra software (LipidAnnotator, MSDial, LipidHunter). Following the criteria applied above, the appropriate number of factors was selected for our data and by regression new variables were created for each of the factors. A hierarchical clustering analysis with squared Euclidean distance and Ward method was applied on the created factors. To select the appropriate number of metabotypes, a discriminant analysis (DA) was performed (Lee et al., 2019).

## Results

1) The presence of heterozygous rare sequence variant in the studied genes, associated to human obesity and energy homeostasis, does not determine different metabolomic phenotypes.

**FIGURE 2:**
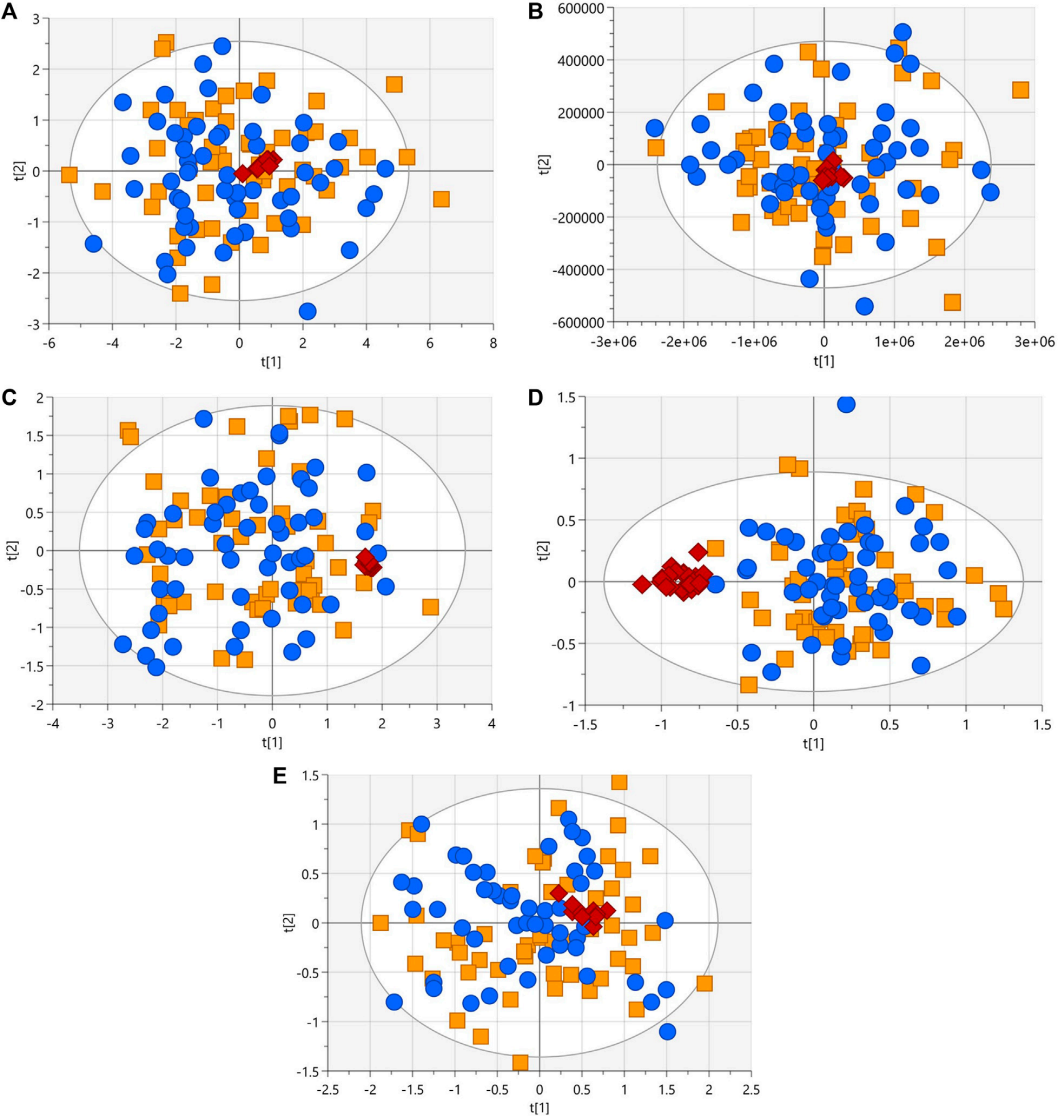
PCA-X score plots (blue dots, patients with heterozygous rare sequence variants (HetRSVs); orange square, patients without variants; red diamonds, QC samples) for the five analytical platforms. **(A)** *R* ^2^ = 0.82, Q^2^ = 0.78 with log10 transformation and Ctr scale (LC-MS (+)). **(B)** *R* ^2^ = 0.937, Q^2^ = 0.594 with Ctr scale (LC-MS (−)). **(C)** *R* ^2^ = 0.516, Q^2^ = 0.458 with log10 transformation and Ctr scale. Four samples were eliminated due to the presence of analytical outliers located outside the hoteling’s ellipse (CE-MS (+)). **(D)** *R* ^2^ = 0.536, Q^2^ = 0.308 with log10 transformation and Ctr scale. Eight samples were eliminated due to the presence of analytical outliers located outside the Hoteling’s ellipse (CE-MS (−)). **(E)** *R* ^2^ = 0.616, Q^2^ = 0.428 with log10 transformation and Ctr scale. Eight samples were removed due to problems during sample preparation (GC-MS).

After following the procedure described in the patients and methods section, we obtained 345 and 170 metabolic features in LC-MS performed in positive and negative ionization modes, 63 signals in GC-MS, and finally in CE-MS we obtained 242 signals in positive ionization and 91 in negative ionization mode. The visual inspection of the PCA plots built for all techniques revealed a tight cluster of the QCs assessing the analytical stability and reproducibility (Figure 2). A homogeneous distribution of patients with and without heterozygous variant was seen in PCA plots.

The identified HetRSVs did not allow for the construction of multivariate supervised model from the results of only one of the analytical techniques. Only LC-MS (+), enabled the creation of an OPLS-DA model (R_2_X = 0.72, R_2_Y = 0.82; Q_2_ = 0.64; p CV-ANOVA_OPLS-DA_ = 3.1 · 10^−19^; as illustrated in Supplementary Figure S1). Correspondingly, the results of three metabolites derived from LC-MS (+) displayed statistically significant differences in means between the variant carrier/no variant groups, whereas no discernible differences between both groups were observed in the means of all the variables from LC-MS (−), GC-MS, CE-MS (+), or CE-MS (−). Furthermore, despite the limited number of samples for each individual gene, the presence of singular metabolic patterns was not observed for any of the studied genes in any of the employed platforms (data not shown).

2) Factor Analysis groups the variability into six factors

Imprecise information is obtained from the examination of the metabolic phenotype using a single analytical platform, which might result in the description of erroneous metabotypes in patients, generating different classifications depending on the analytical platform used (Supplementary Figure S2). Furthermore, the use of a classification based on anthropometric and routine laboratory metabolic and hormonal parameters available in daily clinical practice does not appear to be sufficient to establish distinct metabotype among patients. We also performed a hierarchical clustering analysis, with anthropological and clinical parameters (data not shown). We observed dissimilar outcomes when compared to the classifications produced by individual analytical platforms. Additionally, no statistically significant differences were observed in any of the analytical platforms with the clusters (possible groups) generated after analyzing these parameters.

The multiplatform strategy provided five matrices with 345, 170, 53, 242 and 91 variables analyzed by LC-MS (+), LC-MS (−), GC-MS, CE-MS (+) and CE-MS (−), respectively, from 100 of the studied samples. Due to the presence of analytical outliers caused by errors during sample preparation in GC-MS and analytical error in CE-MS (+), 10 samples had to be eliminated from the analysis of the total of 110 patients enrolled (three patients without genetic variants, and 7 with genetic variants, with a maximum of two individuals per gene studied). As described in detail above (see materials and methods) a Factor Analysis of each of the matrices was performed to subsequently combine the variables present in each Factor Analysis into a single combined Factor Analysis. To eliminate multicollinearity, Pearson correlations were used to analyze the relationships between variables in each matrix (intra-matrix correlations) and between matrices (inter-matrix correlations). In the final Factor Analysis by maximum likelihood and varimax rotation performed on the LC-MS matrix (+), three factors were chosen that explained 75% of the variability accumulated in the matrix, saturating 57 variables that were kept for the final combined Factor Analysis. The Kaiser-Meyer-Olkin (KMO) test was used to determine whether the Factor Analysis was effective, and a result of 0.85 was obtained. In LC-MS (−), two factors were chosen to explain 74.68% of the variability accumulated in the matrix, obtaining a KMO of 0.85 and saturating 17 variables. In GC-MS, three factors were chosen that explain 73.54% of the accumulated variability, obtaining a KMO of 0.89 with 18 variables independently saturated in these factors. In CE-MS (+) 1 factor was chosen that explained 64.09% of the accumulated variation, obtaining a KMO of 0.77 with five saturated variables. In CE-MS (−) no satisfactory factor extraction was achieved, so no variable was retained for the final Factor Analysis. Using in-house databases and mass/mass fragmentation spectra software (LipidAnnotator, MSDial, LipidHunter) all variables that remained after all of these pre-filtering stages for the combined Factor Analysis of the various platforms were identified. The removal of non-annotated variables from the combined Factor Analysis (23 out of 97 variables were removed due to unsuccessful identification) is performed to determine the biological interpretation of the obtained factors. Therefore, variables 43, 11, 14 and 5 analyzed by LC-MS (+), LC-MS (−), GC-MS and CE-MS (+), respectively, were pooled together and the Factor Analysis was performed. The adequacy of the Factor Analysis was tested using the KMO test, obtaining a value of 0.76. Finally, six factors that explained 75% of the accumulated variability were selected. The results show a clustering of the variables into factors depending on the analytical technique. Table 3 shows the variables corresponding to each of the factors (see identification details in Supplementary Table S2). As the resulting factors can be employed to predict discrete clusters of samples, we used all the inferred factors to cluster the patients in the latent factor space, collectively implementing collectively all information from the different analytical platforms.

3) Hierarchical clustering of factors permits to classify patients into metabotypes

**TABLE 3:** Metabolites included in each of the six final factors obtained with their factor scores associated with the factors. Saturations above 0.5 are indicated in dark red. Confidence level in annotation based on Metabolomics Society (Blaženović et al., 2018).

Factor	Identification	Confidence level	Factor score					
			Factor 1	Factor 2	Factor 3	Factor 4	Factor 5	Factor 6
**1**	**SM (d41:2)**	2	0.83	−0.08	−0.06	0.03	0.19	−0.09
**1**	**SM (d42:3)**	2	0.85	0	−0.09	0.05	0.03	−0.08
**1**	**SM (d40:1)**	2	0.83	0.09	−0.07	0.11	0.1	0.07
**1**	**SM (d39:1)**	2	0.72	0	−0.04	0.13	0.24	−0.04
**1**	**SM (d32:1)**	2	0.77	0.07	−0.07	0.02	0.3	0.04
**1**	**SM (d36:2)**	2	0.82	0.22	−0.1	0.15	0	−0.04
**1**	**SM (d34:2)**	2	0.86	0.1	−0.06	−0.03	0.17	0.03
**1**	**SM (d34:0)**	2	0.9	0.09	−0.02	0.11	0.1	−0.03
**1**	**SM (d40:2)**	2	0.87	0.03	−0.08	−0.02	0.24	−0.01
**1**	**SM (d38:1)**	3	0.88	0.16	−0.12	0.1	0.16	−0.01
**1**	**PC (16:0/16:0)**	3	0.78	0.26	−0.03	0.04	0.44	−0.06
**1**	**PC (O-34:1)**	3	0.82	−0.08	0.01	0.03	0.2	−0.06
**1**	**PC (O-40:4)**	3	0.82	−0.03	−0.13	0.2	0.06	−0.06
**1**	**PC (O-32:0)**	3	0.8	0.04	−0.01	0.01	0.17	−0.03
**1**	**PC (O-38:4)**	3	0.81	−0.02	−0.04	0.04	0.17	0.01
**1**	**PC (O-36:5)**	3	0.75	−0.06	0.01	0.01	0.26	0.01
**1**	**PC (16:0/18:2)**	3	0.7	0.28	0.11	−0.14	0.28	0
**1**	**CE (18:2)**	2	0.86	0.11	0.05	−0.1	0.18	0.06
**1**	**CE (20:4)**	2	0.83	0.05	−0.12	0.06	0.15	0.03
**1**	**CE (18:1)**	2	0.83	0.23	−0.03	0.01	0.3	0.02
**2**	**DG (36:4)**	3	0.13	0.81	0.11	0.06	0.23	0.25
**2**	**TG (16:0_18:0_18:1)**	3	0.11	0.77	0.05	−0.1	0.44	0.09
**2**	**TG (56:6)**	3	0.36	0.85	−0.07	0.02	0.14	−0.08
**2**	**TG (18:1_18:2_20:4)**	3	−0.16	0.74	0.07	0.03	0	−0.07
**2**	**TG (56:3)**	3	−0.08	0.83	0.05	−0.04	0.28	0.06
**2**	**TG (58:5)**	3	0.05	0.83	0.02	−0.11	0.29	−0.06
**2**	**TG (56:2)**	3	0.11	0.76	0.02	−0.12	0.3	0.06
**2**	**TG (54:3)**	3	0.21	0.87	0.01	−0.07	0.08	0.05
**2**	**TG (18:1_18:2_18:2)**	3	0.05	0.8	−0.02	−0.15	0.01	−0.04
**2**	**TG (57:2)**	3	0.08	0.85	0.03	−0.09	0.31	−0.02
**2**	**TG (53:3)**	3	−0.06	0.84	0.08	−0.02	0.38	0
**2**	**TG (54:4)**	3	0.09	0.89	0.01	−0.11	−0.03	0
**2**	**TG (16:0_18:1_18:2)**	3	0.19	0.94	−0.02	−0.07	0.13	0
**3**	**Phenylalanine**	2	−0.08	0.02	0.84	0.03	0.05	0.33
**3**	**Oxalic acid**	2	−0.17	−0.08	0.64	−0.1	−0.05	−0.24
**3**	**Myo-Inositol**	2	0.03	0.02	0.65	0.25	0	0.19
**3**	**Cholesterol**	2	0.14	0.15	0.72	0.04	0.06	−0.19
**3**	**Proline**	2	−0.05	0.18	0.78	−0.12	0.14	0.17
**3**	**Serine**	2	−0.02	−0.09	0.93	−0.03	−0.13	0.16
**3**	**Glycine**	2	0	−0.14	0.77	0.05	−0.02	0.15
**3**	**Alanine**	2	−0.06	0.14	0.84	−0.13	0.13	0.11
**3**	**Methionine**	2	−0.09	0.01	0.84	−0.14	0.03	0.11
**3**	**5-Oxoproline/Pyroglutamic acid**	2	−0.12	−0.03	0.81	−0.06	−0.02	−0.08
**3**	**Valine**	2	0	0.15	0.81	−0.01	−0.02	0.05
**3**	**Threonine**	2	−0.03	−0.03	0.85	−0.21	−0.05	−0.01
**4**	**FA (20:4)**	2	0.09	−0.1	−0.03	0.73	−0.08	0.45
**4**	**FA (20:3)**	2	−0.04	0.01	−0.06	0.89	0.04	0.2
**4**	**FA (17:0)**	2	0.04	−0.14	0.05	0.86	−0.05	0.1
**4**	**FA (22:4)**	2	0.07	0	−0.15	0.86	−0.08	0.06
**4**	**FA (18:3)**	2	0.04	0.02	0	0.88	0.11	0.03
**4**	**FA (18:0;O6)**	4	0.04	−0.03	−0.04	0.92	−0.21	0.03
**4**	**FA (14:0)**	2	0.07	−0.03	0	0.87	0.07	0.02
**4**	**FA (22:5)**	2	0.16	−0.08	−0.08	0.91	0.01	−0.01
**4**	**FA (22:6)**	2	0.2	−0.09	0.04	0.64	−0.05	0
**4**	**FA (14:1)**	2	0.04	−0.11	−0.03	0.88	0.01	0
**4**	**FAHFA (2:0_20:4)**	4	−0.06	−0.04	0.02	0.88	−0.22	0.05
**5**	**LPC (20:3/0:0)**	2	0.21	0.18	0.04	−0.02	0.67	0.16
**5**	**PC (18:0_20:3)**	3	0.37	0.33	0.03	−0.06	0.73	0.1
**5**	**PC (30:0)**	3	0.32	0.21	0.05	−0.06	0.76	−0.09
**5**	**PC (16:0_16:1)**	3	0.36	0.3	0.01	0.02	0.78	−0.07
**5**	**PC (34:3)**	3	0.39	0.27	0.03	−0.15	0.79	−0.06
**5**	**PC (40:5)**	3	0.51	0.28	0.1	−0.02	0.69	−0.06
**5**	**PC (33:1)**	3	0.42	0.22	0.03	−0.04	0.68	−0.06
**5**	**PC (18:0_18:1)**	3	0.44	0.21	0.04	−0.07	0.71	0.06
**5**	**PC (18:0_22:4)**	3	0.3	0.28	−0.01	−0.02	0.72	0.03
**5**	**PC (38:1)**	3	0.52	0.18	−0.04	−0.11	0.65	0.01
**6**	**Glutamic acid**	2	−0.04	0.12	0.14	0.18	−0.09	0.85
**6**	**Choline**	2	0.01	0.13	0.17	0.03	0.07	0.74
**6**	**Aspartic acid**	2	0.02	−0.03	−0.01	0.19	0.04	0.72
**6**	**Glutamine**	2	−0.16	−0.1	0.18	0.07	−0.12	0.67
**6**	**Arginine**	2	0.01	0.02	0.12	0.02	0.14	0.66

Bold values means the factor score of each variable in its factor.

**FIGURE 3:**
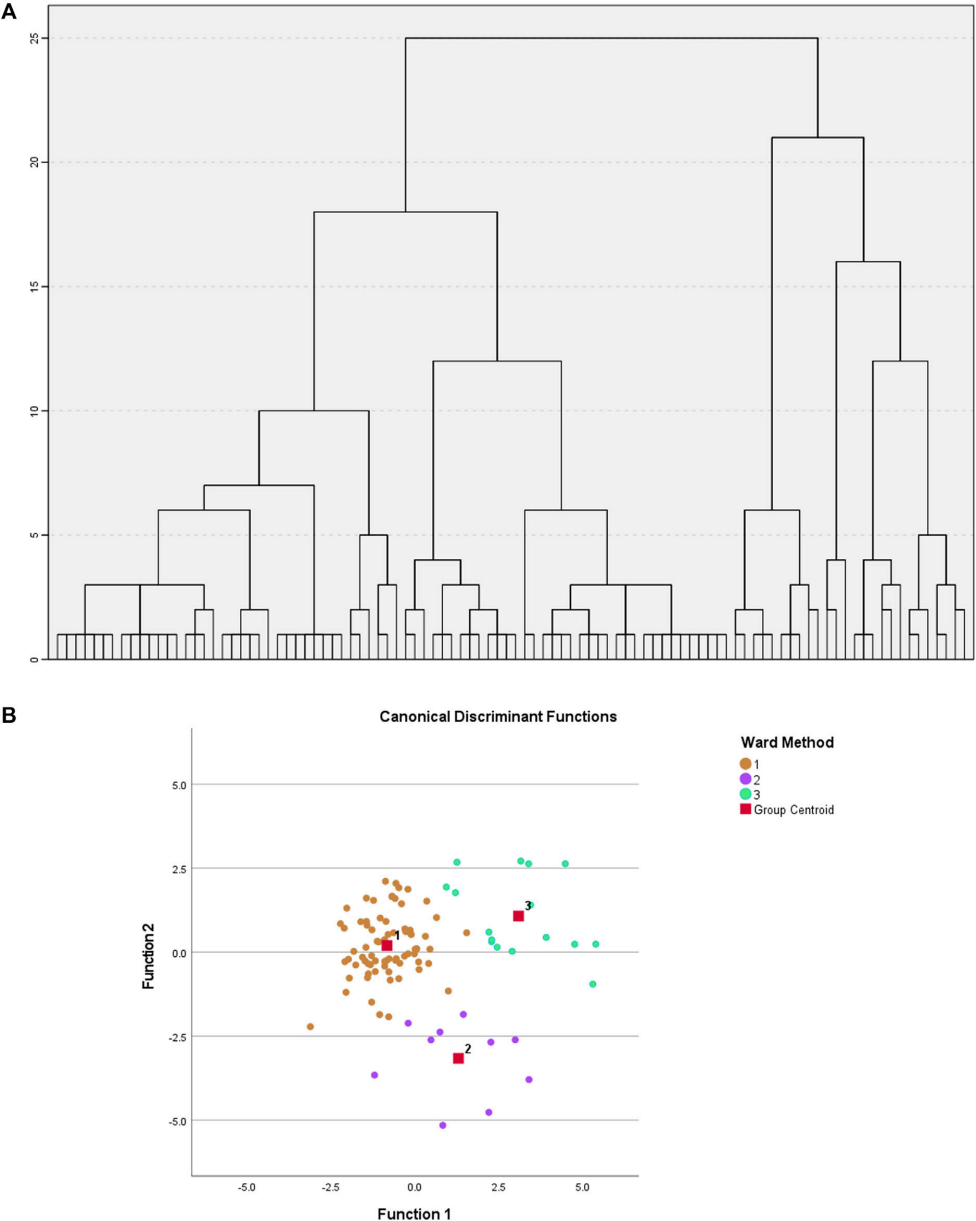
**(A)** Hierarchical clustering performed on the factors obtained after Factor Analysis. Ward’s method and Euclidean distance squared. **(B)** Graph of individuals on the discriminant dimensions. Shows the relative location of the different groups.

A hierarchical clustering analysis with Ward method and squared Euclidean distance was applied in SPSS statistical software (Figure 3A). To determine the optimal number of metabotypes, a discriminant was applied to 2, 3 and 4 metabotypes. It appears that grouping the samples into three distinct metabotypes provides the most robust explanation for the observed relationships, where 94% accuracy was observed after cross-validation, demonstrating the existence of 3 clearly differentiated metabotypes [metabotype 1 (G1) (*n* = 74), metabotype 2 (G2) (*n* = 10), metabotype 3 (G3) (*n* = 16)] (Figure 3B).

The identified factors enabled metabotypes to be characterized. The components of the first two factors, F1 (10 sphingolipids, 4 ether-linked phosphatidylcholines, 2 phosphatidylcholines and 3 cholesterol esters, named “*Lipids1*”) and F2 (1 di- and 12 tryacylglycerols, named “*Lipids2*”), accounted for 44% of the variability and were increased in metabotype 3 and decreased in metabotype 1. F3 components (named “*nutritional amino acids*,” including eight amino acids) showed increased levels in metabotype 2. F4 elements (including nine circulating free fatty acids, named “*Lipids3*”) showed increased levels in metabotype 1. F5 elements (including 10 phosphatidylcholines, named “*Lipids4*”) and the components in F6 (glutamic acid, choline, aspartic acid, glutamine, and arginine, named “*signaling amino acids*”) showed an accumulated variation of 75% and were increased in metabotype 3.

**FIGURE 4:**
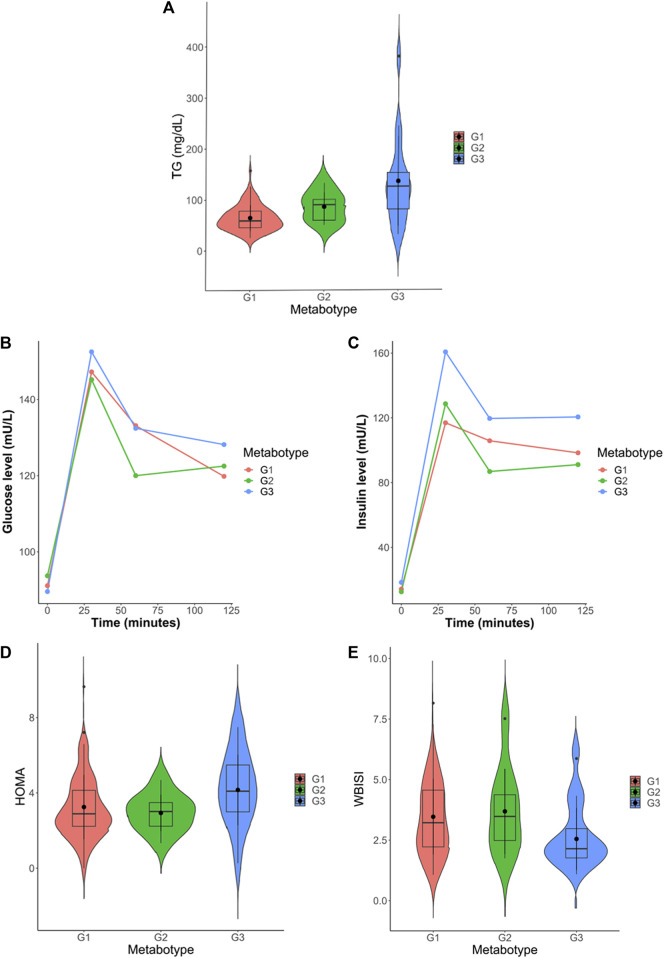
**(A)** Violin plot of total triglyceride levels in the three identified metabotypes. **(B)** Progression plot of insulin levels throughout the oral glucose tolerance test (OGTT). **(C)** Progression plot of glucose levels throughout the oral glucose tolerance test (OGTT). **(D)** Violin plot of HOMA-IR (Homeostatic Model Assessment for Insulin Resistance) levels in the three identified metabotypes. **(E)** Violin plot of WBISI (whole-body insulin sensitivity index) levels in the three identified metabotypes.

Univariate statistics were performed on each of the matrices. Each variable was analyzed by ANOVA or its corresponding non-parametric method (Kruskal–Wallis). The overall results show the greatest differences between metabotype 1 and metabotype 3 (208 metabolites with p-Bonferroni< 0.05 out of a total of 964 variables) mainly in triglyceride, diglyceride and phosphatidylcholine levels. In addition, there are also differences (125 metabolites) between metabotype 2 and metabotype 3. Only one of these 125 significant metabolites is different from the comparison between metabotype 1 and metabotype 3, this was lactic acid. However, differences between metabotype 1 and metabotype 2 are minimal (7 metabolites). It is important to note that the levels of different triglycerides in metabotype 3 are found to be increased over two-fold over those in metabotypes 1 and 2. In addition, we observed a significant reduction of proline in metabotype 1. Intergroup comparison of routine clinical laboratory data revealed significant differences in total triglyceride levels, along with fasting and glucose-stimulated serum insulin but not glucose level among these metabotypes (Figure 4; Table 4), with individuals in metabotype 3 showing lower insulin sensitivity and hypertriglyceridemia, in a higher risk metabolic profile than patients in metabotypes 1 and 2.

**TABLE 4:** Clinical/biochemical parameters in the studied metabotypes. Values are average ± SEM. *p*-value was computed according to the parametric or non-parametric tests applied (ANOVA/Kruskal–Wallis), selected accordingly. Groups homogeneity (Bonferroni) is indicated with superscript letters. Shared letter involves homogeneous groups.

Clinical features	G1	G2	G3	*p*-value
Age (years)	10.8 ± 0.4	12 ± 0.9	11.8 ± 1	0.34
BMI (kg/m^2^)	28.4 ± 0.5	28 ± 0.9	29.8 ± 1.5	0.65
BMI-SDS	4.2 ± 2.4	3.6 + 1.0	3.9 + 1.6	0.87
HOMA	3.2 ± 0.2	2.9 ± 0.3	4.2 ± 0.5	0.08
Insulin levels 0 min (µU/mL)	14.2 ± 0.8^a^	12.6 ± 1.2^ab^	18.4 ± 1.9^b^	**0.03**
Insulin levels 30 min (µU/mL)	116.9 ± 8^a^	128.7 ± 24.5^ab^	160.7 ± 17.8^b^	**0.03**
Insulin levels 60 min (µU/mL)	105.8 ± 9.3	86.9 ± 24.7	119.6 ± 14.7	0.17
Insulin levels 120 min (µU/mL)	98.4 ± 8.6	91.1 ± 17.9	120.6 ± 18.1	0.36
WBISI	3.5 ± 0.2	3.7 ± 0.6	2.6 ± 0.3	**0.05**
Glucose levels 0 min (mg/dL)	91.1 ± 0.8	93.7 ± 1.9	89.6 ± 1.9	0.32
Glucose levels 30 min (mg/dL)	147.2 ± 2.9	145.2 ± 7.7	152.5 ± 5.9	0.68
Glucose levels 60 min (mg/dL)	133.1 ± 3.3	120 ± 5.9	132.4 ± 5.7	0.35
Glucose levels 120 min (mg/dL)	119.8 ± 2	122.5 ± 5.1	128.1 ± 5	0.24
Total Cholesterol (mg/dL)	154.2 ± 3.7^a^	160.9 ± 8.9^ab^	178.9 ± 9.9^b^	0.03
TG (mg/dL)	64.2 ± 2.9^a^	85.5 ± 8.9^ab^	135.3 ± 22^b^	**0.00**
HDL-c (mg/dL)	48 ± 1.8	45.3 ± 3.4	41.4 ± 2.2	0.19
LDL-c (mg/dL)	92.7 ± 3.2	99.5 ± 7	110.5 ± 7.6	0.19
GGT (U/L)	14 ± 0.5	15 ± 1.6	15.9 ± 1.3	0.35
GOT (U/L)	27.4 ± 0.8	26.9 ± 1.4	30.1 ± 2.7	0.87
GPT (U/L)	21.2 ± 0.9	23 ± 3.7	26.9 ± 3.4	0.13
IGF-1 (ng/mL)	320.3 ± 19.9	289.4 ± 41.3	293 ± 49.5	0.64
IGFBP3 (µg/mL)	4.9 ± 0.1	4.6 ± 0.3	5.1 ± 0.3	0.52
HbA1c (%)	5.4 ± 0	5.5 ± 0.1	5.3 ± 0.1	0.58
Free T4 (ng/dL)	0.9 ± 0.1	1.0 ± 0.1	0.9 ± 0.1	0.61
TSH (µU/mL)	2.7 ± 0.2^ab^	1.8 ± 0.3^a^	3.3 ± 0.5^b^	**0.03**
DBP mmHg	61.7 ± 0.9	62.2 ± 2.1	64.7 ± 1.9	0.22
SBP mmHg	116.3 ± 1.5	115.6 ± 3.7	123.1 ± 3.5	0.17
Uric Acid mg/dL	5.2 ± 0.1	5.0 ± 0.4	5.3 ± 0.3	0.76
Vitamin D (ng/mL)	24.4 ± 1.3	23.1 ± 3.8	20.6 ± 2.8	0.28

Abbreviations: BMI-SDS, Standardized body mass index (Z-score); HOMA, homeostatic model assessment; WBISI, Whole-body insulin sensitivity index; TG, triglycerides; HDL-c, High density lipoprotein cholesterol; LDL-c, Low density lipoprotein cholesterol; GGT, Gamma-glutamyltransferase; GOT, aspartate transaminase; GPT, glutamic-pyruvic transaminase Alanine aminotransferase; IGF-1, Insulin-like growth factor 1; IGFBP3, Insulin-like Growth Factor-binding Protein 3; HbA1c: hemoglobin A1c; TSH, thyroid-stimulating hormone; DBP, diastolic blood pressure; SBP, diastolic blood pressure.

## Discussion

In this study, we have highlighted the significance of conducting an in-depth analysis of individuals’ metabolic phenotypes, yielding a classification that cannot be attained through anthropometric features or routine clinical laboratory analyses. Furthermore, we have observed an absence of pathognomonic metabolic or metabolomics signatures due to the presence of specific HetRSVs. In this context, Factor Analysis assumes particular importance in the integration of data from various analytical platforms, bringing us closer to personalized medicine.

The term “personalized medicine” stands for the most suitable specific therapeutic interventions for an individual patient, underscoring the relevance of developing management strategies on specific individuals and not average group response to treatments. This concept has also expanded to nutrition (i.e., personalized nutrition) and current research focuses on the intricate interaction between diet, (epi)genome, and the microbiome, which can determine the effects of bioactive compounds (González-Sarrías et al., 2017).

Using a multiplatform untargeted metabolomics-based approach, we determined the metabolic fingerprint of children with obesity, and by integrating all the data generated by using Factor Analysis to stratify individuals with obesity according to their metabolic phenotype, we defined three different “metabotypes.” Bioinformatics tools are currently available to combine information from different omics technologies or from different analytical platforms. Some of these tools allow the performance of supervised multivariate analysis (Westerhuis et al., 1998; Löfstedt and Trygg, 2011; Boccard and Rutledge, 2013) to determine the existing differences between different groups combining the obtained data. Other integrative multi-omics clustering tools are specific unsupervised integrative methods to find coherent groups between samples or features using the information obtained in a multi-omics analysis (Multiblock PCA, iClusterPlus, iClusterBayes, moCluster, LRAcluster, PINSplus, SNF, etc.) (Mo et al., 2013; Wu et al., 2015; Meng et al., 2016; Nguyen et al., 2017; Wang et al., 2017; Mo et al., 2018; Rappoport and Shamir, 2018; Nguyen et al., 2019; Tanabe et al., 2021; Zhang et al., 2022). However, most of these algorithms require knowledge about the parameters to be applied, and some exhibit complex interpretability. The advantage of Factor Analysis is that it allows us to reduce dimensionality (without losing statistically relevant information), which facilitates the discovery of potential biomarkers, as well as simplifies the biological interpretation of differences between individuals’ metabolic phenotypes. Data integration based on dimensionality reduction approaches seems to be a powerful tool to combine all metabolomic information obtained from different platforms (Zhang et al., 2022). This study proposes the use of Factor Analysis to combine and summarize the information from the different data matrices. The use of Factor Analysis combined with a hierarchical clustering analysis has made it possible to identify three clearly differentiated metabotypes between children with obesity. It is known that cluster analysis has the potential to yield clusters that are either arbitrary or devoid of biological significance. One strength of the results obtained relies on the fact that the acquisition of a notably elevated score in a supervised analysis (discriminant analysis) employing the metabotypes derived from the cluster analysis, serves to not only validate the efficacy of the Factor Analysis but also to enhance the concrete manifestation of the three identified metabotypes.

In routine clinical laboratories, serum levels of triglycerides, lipoproteins, and transaminases are frequently increased in patients with obesity, revealing underlying dyslipidemia and liver dysfunction (Rauschert et al., 2016). Several studies indicate that some amino acids, such as the branched chain amino acids (BCAA), tyrosine, valine, leucine, or isoleucine, can be used as indicators in early stages of carbohydrate metabolism impairment (Wang et al., 2011; Michaliszyn et al., 2012; Mccormack et al., 2013; Butte et al., 2015; Mastrangelo et al., 2016; Suzuki et al., 2019). Moreover, Suzuki et al. reported a correlation between insulin resistance and free amino acid levels in a cohort of patients with moderate to severe obesity (Suzuki et al., 2019). Our results suggest the existence of large metabolic differences between the identified metabotypes, with a singularly differentiated fingerprint in metabotype 3. Factor Analysis indicates that metabotype 3 is characterized by increased levels of “*Lipids1*,” “*Lipids2*,” “*Lipids4*,” and amino acids related to cell signaling. In addition, univariate analysis showed mainly significant differences in triglycerides, diglycerides, and phosphatidylcholines between metabotype 3 and the rest of the metabotypes, with increased levels of these lipid species in metabotype 3. These results suggest the presence of combined hyperlipidemia (cholesterol + triglyceride) in individuals integrated within metabotype 3. Routine clinical laboratory analyses are partially in agreement with these results as individuals in metabotype 3 had increased total triglyceride levels, as well as impairment of insulin, including increased fasting and glucose stimulated insulin secretion and lower WBISI, along with significantly increased levels of isoleucine and proline (UVA, data not shown), in concordance with Suzuki et al. (Suzuki et al., 2019). However, routine clinical analysis of cholesterol species did not detect the higher cholesterol ester levels in metabotype 3 observed by using metabolomics, even when other studies have associated increased cholesterol and triglyceride levels with a decrease in HDL-c levels (Brown et al., 2000) and higher BMI (Huynh et al., 2019). It is pertinent to emphasize that the more pronounced metabolic perturbation of individuals in metabotype 3 is not correlated with a higher BMI-SDS among these individuals compared to those in other metabotypes. Nevertheless, a higher representation of Hispanic ethnicity was observed in metabotype 3 (25%) compared to metabotypes 1 (12%) or 2 (0%). This is consistent with the lower insulin sensitivity and higher triglyceride levels reported in Hispanic children with obesity compared to Caucasians (Martos-Moreno et al., 2020), thus suggesting an eventual ethnic driven influence in obesity associated metabolomic profiles (Butte et al., 2015), although this does not extend to all difference observed (i.e., higher cholesterol ester levels in metabotype 3, not endorsed in inter-ethnic comparisons) (Martos-Moreno et al., 2019; Martos-Moreno et al., 2020). In contrast, proven the role of pubertal status on the development of obesity associated metabolic comorbidities (particularly insulin resistance), we compared the relative frequence within each defined metabotype of patient Tanner stage and, additionally, of prepubertal vs. pubertal patients (pooling TII to T-V in the latter). No significant differences between metabotypes were observed regarding the distribution of Tanner stages within each metabotype (χ2 0.491; *p* = 0.782) nor in the relative proportion of prepubertal vs. pubertal patients (χ2 8.596; *p* = 0.378). Despite these results not being supportive, the possibility of pubertal influence on the patient metabotype cannot be completely ruled out and this should be further explored in larger patient cohorts.

Interestingly, Factor Analysis splits the relevant amino acids into two subsets. Phenylalanine, proline, serine, glycine, alanine, methionine, valine, and threonine were part of Factor 3, and were higher in Metabotype 2. Glutamic acid, glutamine, and arginine, together with choline were higher in Metabotype 3. Even though these amino acids have all been shown to correlate with insulin resistance in childhood obesity (Suzuki et al., 2019), such grouping points towards a non-homogeneous involvement of the different amino acids in the complications of obesity. Besides their role in protein synthesis, each amino acid can be involved in different functions and processes, and it is beyond the possibilities of this observational study to determine the exact relationships between the differences found and the therapeutic approach to treat obesity. Those amino acids grouped in Factor 3 include 4 essential (phenylalanine, threonine, valine, methionine) and 3 of the most abundant amino acids (glycine, serine, alanine), and therefore this factor could be strongly related to the nutritional status of the patients, as it would represent protein intake and turnover in the body. In Factor 6, increased in Metabotype 3, glutamic acid, aspartic acid and glutamine, were grouped with choline and arginine. In addition to also reflecting the nutritional status, this group of factors is of particular relevance as they can be related to neurotransmission (Dalangin et al., 2020), and their circulating levels have been proposed as biomarkers of visceral obesity and metabolic alterations (Maltais-Payette et al., 2018) and have been associated with metabolic stress (Yan et al., 2012).

As stated above, childhood obesity is the result of the action of multiple environmental factors on eating and activity habits and lifestyle, in combination with an individual’s unique genetic fingerprint. GWAS studies yielded a large list of genes with SNPs, or variants associated to human obesity, but in the vast majority of cases, a single determinant of childhood obesity cannot be identified, thus classifying these cases as “polygenic” or “idiopathic” obesity. In contrast, the rare cases of monogenic forms of obesity, are mainly caused by biallelic mutations in a single gene, usually in the leptin-melanocortin satiety pathway, and are characterized by vary severe, early-onset obesity, usually with evident hyperphagia, and in some cases associated to other metabolic comorbidities and influencing growth pattern even in the first years of life (Handakas et al., 2022). Several metabolomic studies have been performed in childhood obesity, comprehensively characterizing the metabolic alterations in these conditions, as well as in animal models of leptin resistance thus exploring the effect of the impairment of the leptin-POMC satiety pathway (Pietiläinen et al., 2007; Mastrangelo et al., 2016; Martos-Moreno et al., 2017; Rauschert et al., 2017; Kim et al., 2019; Lawler et al., 2020; Rupérez et al., 2020; Sanz-Fernandez et al., 2020). However, the pathogenic role of heterozygous rare sequence variants in the genes of the leptin-melanocortin pathway (Le Collen et al., 2023), as in other genes relevant for central energy and glucose homeostasis is under discussion (Trang and Grant, 2023). Following previous observations by us and other groups (Serra-Juhé et al., 2017; Gerl et al., 2019; Gonzalez-Riano et al., 2021), the hypothesis that they are eventual pathogenicity was proposed. However, the results of this study, showing no anthropometric, metabolic nor metabolomic differences between patients with or without HetRSVs in the studied genes, and the lack of differences in the prevalence of the different metabotypes between these groups does not verify their pathogenic role (Supplementary Tables), at least from a metabolic and metabolomic point of view. To our knowledge, this study is the first metabolomic study attempting to identify a specific metabolic phenotype associated with the presence of HetRSVs in the leptin-POMC pathway, as well as in glutamate receptors, and demonstrates the absence of a clear differential metabolic phenotype due to the presence of these variants.

The association of obesity with lipidomes and the use of technologies to stratify obesity based on lipidomic data has been previously investigated by means of machine learning algorithms (Gerl et al., 2019). However, this is the first study, to combine metabolomic data from different analytical platforms and genetic data to stratify obesity. Moreover, Factor Analysis has not been previously employed for the subclassification of patients with obesity by using the adequate combination of multiplatform MS metabolomics data. We distinguished two antagonic metabotypes (1 and 2 vs. 3) that can be deduced from the examination of the contributing factors. Such subclassification was not possible from the information derived from the routine clinical examination and laboratory analyses. With our approach, subtle but clear differences arose between the three metabotypes: Six groups of metabolites can be combined to evaluate the metabolic phenotype, and promising associations between this metabolic phenotype and insulin sensitivity, circulating triglycerides and TSH levels and ethnicity have been uncovered. Metabotypes 1 and 2 have lower levels of the factors corresponding to “*Lipids1*” (F1), “*Lipids2*” (F2), “*Lipids4*” (F5) and “*Signaling amino acids*” (F6), as compared to Metabotype 3, suggesting a higher metabolic risk phenotype in patients with childhood obesity in Metabotype 3 (Table 5). Among the most promising results, the separation of amino acids into two different factors, and the differential association of these factors with different phenotypes opens the possibility of treating the obese subjects in these two metabotypes with different approaches. Metabotype 2 was associated with higher levels of F2, or *Nutritional amino acids*, than in the other two metabotypes, suggesting a healthier metabolic phenotype of these patients, that could speculatively be associated to higher protein intake in their diet, whereas F6, higher in metabotype 3, could speculatively be associated to a behavioral component of the children in this group. However, the lack of precise control of feeding behavior in these patients, feeding due to the ambulatory modality of management is a limitation to test this hypothesis. Apart from this, the limited number of patients studied, along with the potential confounding factors (such as sex, race or pubertal status) potentially influencing the described metabotypes raise the need of validating the presented results in larger and independent cohorts, to enhance the reliability and generalizability of the results, i.e., to support the metabotypes here identified and to explore an eventual role of these factors.

**TABLE 5:** Mean values of the factor scores of the new factors obtained after Factor Analysis.

Factor name		G1	G2	G3
		Mean	Mean	Mean
Factor 1	“*Lipids1*” (SM/CE)	−0.06	−0.03	0.32
Factor 2	“*Lipids2*” (DAG/TAG)	−0.30	0.01	1.38
Factor 3	“*Nutritional amino acids*”	−0.33	2.10	0.20
Factor 4	“*Lipids3*” (FFA)	0.05	−0.13	−0.16
Factor 5	“*Lipids4*” (PC)	−0.15	−0.29	0.88
Factor 6	“*Signaling amino acids*”	−0.08	−0.59	0.75

The challenge for the near future will be to use new technological advances such as that used here to accurately stratify the state/stage of different diseases, in order to precisely predict disease progression and to provide appropriate treatment for each patient, as well as to monitor their evolution.

## Data Availability

This study is available at the NIH Common Fund’s National Metabolomics Data Repository (NMDR) website, the Metabolomics Workbench, https://www.metabolomicsworkbench.org where it has been assigned Study ID ST002993. The data can be accessed directly via its Project DOI: http://dx.doi.org/10.21228/M8WX4S. This work is supported by NIH grant U2C-DK119886. The study is available for review at http://dev.metabolomicsworkbench.org:22222/data/DRCCMetadata.php?Mode=Study&StudyID=ST002993&Access=ZeeZ9799.
