# Supplementary material for: Identifying subgroups of childhood obesity by using multiplatform metabotyping
Source: Front Mol Biosci. 2023 Dec 20;10:1301996. doi: 10.3389/fmolb.2023.1301996 (PMC10761426; doi:10.3389/fmolb.2023.1301996)
Supplement: Supplementary file 1 [file DataSheet1.docx]

**IDENTIFYING SUBGROUPS OF CHILDHOOD OBESITY BY USING MULTIPLATFORM METABOTYPING**

David Chamoso-Sanchez^1^, Francisco Rabadán Pérez^4^, Jesús Argente^2,3,5^, Coral Barbas^1^, Gabriel A. Martos-Moreno^2,3*^, Francisco J. Rupérez^1*^

1 Centro de Metabolómica y Bioanálisis (CEMBIO), Facultad de Farmacia, Universidad San Pablo-CEU, CEU Universities, Urbanización Montepríncipe, 28660 Boadilla del Monte. España

2 Departments of Pediatrics & Pediatric Endocrinology, Hospital Infantil Universitario Niño Jesús, 28009, Madrid, Universidad Autónoma de Madrid. Madrid, España

3 CIBER fisiopatología de la obesidad y nutrición (CIBEROBN). Instituto de Salud Carlos III. Madrid, España

4 Universidad Rey Juan Carlos, Departamento Economía Aplicada I.

5 IMDEA Food Institute. Madrid. España.

**KEYWORDS:** Multiplatform metabolomics, Factor Analysis, Data integration, Obesity, Childhood, Leptin-Melanocortin pathway.

Francisco J.Rupérez*

[ruperez@ceu.es](mailto:ruperez@ceu.es)

Gabriel A.Martos-Moreno*

[gabrielangelmartos@yahoo.es](mailto:gabrielangelmartos@yahoo.es)

**SUPPLEMENTARY NOTE 1**

**Chemicals**

Using a Mili-Qplus185 system (Milipore, Billerica, MA, SA) we prepared the aqueous solutions using reverse-osmosed ultrapure water obtained “in-house” to prepare all buffers and standard solutions. Acetonitrile (ACN), isopropanol (IPA) and LC-MS grade methanol (MetOH) were purchased from Fisher Scientific (Pennsylvania, United States). Acetic acid glacial (AnalaR® NORMAPUR®) and analytical grade ammonia solution (28%, GPR RECTAPUR®) were obtained from VWR Chemicals (Pennsylvania, United States), and ammonium fluoride (NH_4_F) (ACS reagent, ≥98%) was obtained from Sigma-Aldrich (Steinheim, Germany). Chloroform from ROMIL Pure Chemistry (Cambridge, United Kingdom) and tert-Butyl methyl ether (MTBE) from Sigma Aldrich (Steinheim, Germany). The internal standards (IS) used in LC-MS were palmitic acid-d31 from Sigma-Aldrich (Steinheim, Germany) and Sphinganine (D17:0) from Avanti Polar Lipids, INC (Birmingham, United States), in CE-MS were methionine sulfone, paracetamol and Morpholineethanesulfonic acid, 2-(N-Morpholino)ethanesulfonic acid (MES) from Sigma Aldrich (Steinheim, Germany), in GC-MS were palmitic acid-d31 and tricosane from Sigma Aldrich (Steinheim, Germany). Reagents for derivatization in GC-MS (*O*-methoxyamine hydrochloride and BSTFA: TMCS, 99:1 (Sylon BFT)) were purchased from Sigma-Aldrich. Two standard mixes for GC-MS, one containing grain fatty acid methyl esters (C8:0-C22:1, n9) and another standard mix with a mixture of *n*-alkanes (C8-C40) were obtained from Fluka Analytical (Sigma-Aldrich Chemie GmbH, Steinheim, Germany). Finally, n-heptane, 99% and sylilation-grade pyridine were obtained from Carlo Erba Reagents-SA (DASITGROUP, Spain) and AnalaR® NORMAPUR® (VWR Chemicals, Pensylvannia, United States).

**Metabolites extraction for Gas Chromatography coupled with mass spectrometry analysis.**

The 110 randomly selected serum samples (monogenic childhood obesity group, n=55; idiophatic childhood obesity group, n=55) were thawed on ice approximately 1 hour. The samples were vortex-mixed for 2 minutes and 40 µL of serum were transferred to an Eppendorf tube. A volume of 120 µL of cold acetonitrile (-20 ºC) with palmitic acid-d31 (25 ppm) as internal standard was used for deproteinization. Then, they were vortex-mixed for 2 minutes, incubated on ice and centrifuged for 10 minutes at 15,400xg at 4ºC. Sample preparation continued with 100 µL of the supernatant were transferred to a GC-MS vial to be evaporated to dryness in a vacuum concentrator. When the vials were completely dry, the derivatization process was continued to obtain volatile derivatives for analysis using MultiPurpose Autosampler for GC - GC/MS from GERSTEL (Linthicum, United States). First, 20 µL of *O*-methoxyamine in pyridine (15mg/mL) were added to each of the vials. The samples were then vortex-mixed vigorously for 10 minutes with a stirrer speed of 1000 rpm at room temperature. Then, the samples were incubated at 60 ºC for 90 minutes with a stirrer speed of 750 rpm. The samples were kept at standstill for 5 min and then 40 µL of BSTFA: TMCS (99:1) and vortex-mixed for 10 minutes with a stirrer speed of 1000 rpm at room temperature. The samples were incubated at 60ºC for 90 minutes with a stirrer speed of 750 rpm at 60 ºC. Then, the samples were kept at standstill for 30 minutes at 8ºC and 80 µL of heptane with 20 ppm of tricosane (internal standard) were added to the samples and mixed for 5 minutes in the vortex mixer at 1000 rpm. Finally, the samples were kept at standstill for 30 minutes at 8ºC and 2 µL were injected into the system.

**Metabolites extraction for Liquid chromatography coupled with mass spectrometry (LC-QTOF-MS) analyses.**

The 110 randomly selected serum samples (monogenic childhood obesity group, n=55; idiopathic childhood obesity group, n=55) were thawed on ice approximately 1 hour. The samples were vortex-mixed for 2 minutes and 40 µL of serum were transferred to and Eppendorf tube. Subsequently, 800 µL of a previously prepared cold mixture (-20 ºC) of methanol:MTBE:Chloroform (1.33:1:1, v/v/v) with Sphinganine (D17:0) and palmitic acid-d31 as internal standards were added in the Eppendorf tube for desproteinization. They were vortex for 30 seconds and were shaken 20 min at maximum speed at room temperature. Samples were centrifuged for 5 minutes at 13,200 rpm at 4 ºC. After centrifugation, the supernatant was transferred to a chromatography vial with insert and was directly injected into the system.

**Metabolites extraction for capillary electrophoresis coupled with mass spectrometry analysis.**

After thawing the samples on ice for 1 hour, they were vortex-mixed for 1 minute and 80 µL of serum were transferred to an Eppendorf tube. Subsequently, 80 µL of a previously prepared cold mixture (-20 ºC) of 0.2 M formic acid containing 5% acetonitrile and as internal standard 0.4 mM methionine sulfone, 2 mM paracetamol and 0.5 mM 4-morpholineethanesulfonic acid, 2-(N-morpholino)ethanesulfonic acid (MES) were added. The samples were vortex-mixed for 1 minute and was transferred to a centrifree ultracentrifugation device (Millipore Ireland Ltd., Carrigtohill, Ireland) with a 30 kDa protein cutoff for deproteinization through centrifugation (2000×g, 4 °C, 90 min). After centrifugation, 90 µL of the supernatant were transferred to a CE-MS vial for analysis.

**Analytical settings for the UHPLC-QTOF-MS**

Serum lipidomics was performed using an UHPLC system (1290 Infinity II Bio LC System, Agilent Technologies, Waldbronn, Germany), coupled to a 6545 Liquid Chromatography – Quadrupole – Time of Flight Mass Spectrometer mass spectrometer (LC/Q-TOF MS) (Agilent Technologies) with an ESI ion source. Samples were analyzed following the previous described method by Carolina et al (1). Briefly, the Agilent 1290 Infinity II Multisampler system, equipped with a multiwash option, was used to uptake 1 μL of extracted samples. The temperature of the multisampler was maintained at 15 ºC to avoid lipid precipitation and preserve compounds in a stable environment. An Agilent InfinityLab Poroshell 120 EC –C18 (3.0 ×100 mm, 2.7 μm) (Agilent Technologies) column and a compatible guard column (Agilent InfinityLab Poroshell 120 EC –C18, 3.0 ×5 mm, 2.7 μm) were used and maintained at 50 °C. The mobile phases used for both positive and negative ionization modes consisted of (A) 10 mM ammonium acetate, 0.2 mM ammonium fluoride in water/methanol (9:1) and (B) 10 mM ammonium acetate, 0.2 mM ammonium fluoride in acetonitrile/methanol/isopropanol (2:3:5). The chromatography gradient started at 70% of B at 0-1 min, 86% B at 3.5-10 min, 100% B at 11-17 min. The starting conditions were recovered by minute 17, followed by a 2 min re-equilibration time; the total running time was 19 min. The flow rate was held constant, set at 0.6 mL/min. The multiwash strategy consisted of a mixture of methanol: isopropanol (50:50, v/v) with the wash time set at 15 s, and aqueous phase:organic phase (30:70, v/v) mixture to assist in the starting conditions (1).

The Agilent 6545 QTOF mass spectrometer equipped with a dual AJS ESI ion source was set with the following parameters: 150 V fragmentor, 65 V skimmer, 3500 V capillary voltage, 750 V octopole radio frequency voltage, 10 L/min nebulizer gas flow, 200 °C gas temperature, 50 psi nebulizer gas pressure, 12 L/min sheath gas flow, and 300 °C sheath gas temperature. Data were collected in positive and negative ESI modes in separate runs, operated in full scan mode from 40 to 1200 m/z with a scan rate of 3 spectra/s. We use two reference mass compounds throughout the whole analysis: purine (C_5_H_4_N_4_) at m/z 121.0509 for the positive and m/z 119.0363 for the negative ionization modes; and HP-0921 (C_18_H_18_O_6_N_3_P_3_F_24_) at m/z 922.0098 for the positive and m/z 980.0163 (HP-0921 + acetate) for the negative ionization modes. These masses were continuously infused into the system through an Agilent 1260 Iso Pump at a 1 mL/min (split ratio 1:100) to provide a constant mass correction. Ten Iterative-MS/MS runs were performed for both ion modes at the end of the analytical run, five iterative-MS/MS runs were set with a collision energy of 20 eV, and the subsequent five runs were performed at 40 eV. They were operated with an MS and MS/MS scan rates of 3 spectra/s, 40–1200 m/z mass window, a narrow (∼1.3 amu) MS/MS isolation width, 3 precursors per cycle, and 5000 counts and 0.001% of MS/MS threshold. References masses were excluded from the analysis to avoid inclusion in the iterative-MS/MS. Data was acquired using Agilent MassHunter Workstation Software LC/MS Data Acquisition for 6200 series TOF/6500 series Q-TOF B 9.0.9044.0 (Agilent Technologies).

**Analytical setting for the GC-MS analysis**

A MultiPurpose Autosampler for GC - GC/MS from GERSTEL (Linthicum, United States) coupled to an 8890 GC System coupled to a 5977B GC/MSD mass spectrometer with a single quadrupole from Agilent Technologies (Waldbronn, Germany) was used to perform metabolite of serum samples. Briefly, 2 µL of derivatized samples were automatically injected in split mode (ratio 1:10). The separation of the compounds was achieved using a pre-column (10 m J&W integrated with Agilent 122‐5532G) combined with a GC DB5-MS column (length, 30 m; internal diameter, 0.25 mm; and 0.25 μm film of 95% of dimethyl/5% diphenylpolysiloxane). The total analysis time for each sample is 37 minutes, with 5 minutes post run time. The flow rate of the carrier gas (helium) was constant at 0.5508 mL/min through the column. The temperature of the column was initially set at 60 °C for 1 minute, then raised to 10 °C/min to 325 °C, which was maintained for 10 minutes before cooling. The injector and transfer line temperatures were set at 250 °C and 280 °C, respectively. The operating parameters of electronic impact ionization were established as follows: filament source temperature at 230 ° C and electronic ionization energy at 70 eV. Mass spectra were collected in a mass range of 50 to 600 m/z at a scan rate of 2 spectra per second. Data was acquired using Agilent MassHunter Workstation GC/MS Data Acquisition B 10.0.384.1 software (Agilent Technologies). To determine the retention rate, a mixture of n-alkanes (C8-C28) dissolved in n-hexane was analyzed before the samples.

**Analytical setting for the CE-MS analysis**

A 7100 capillary electrophoresis (Agilent Technologies) coupled to a 6230 time-of-flight mass spectrometer (TOF MS) (Agilent Technologies), equipped with an electrospray ionization (ESI) source was used to perform metabolite of serum samples in positive ionization. An Agilent Technologies fused silica capillary (total length, 100 cm; internal diameter, 50 µm) was used for the separation of metabolites working in normal polarity. Before each analysis, the capillary was washed for 5 min (950 mbar) with background electrolyte (BGE) (1 M formic acid solution in 10% methanol (v/v)). Samples injection was performed during 50 s at 50 mbar and, to improve the analysis reproducibility, BGE was injected for 20 s at 100 mbar after each sample injection. The separation was performed with an internal pressure of 25 mbar at a voltage of 30 KV and at a constant temperature of 20 ° C, and the current observed under these conditions was 50 μA. The total analytical run time was 26 minutes. Mass spectrometry was operated in positive polarity, with a mass range 70–1000 m/z at a rate of 1.36 spectrum /s. Other parameters for the MS were: fragmentor at 125 V, skimmer at 65 V, OctopoleRFPeak at 750 V, drying gas temperature at 200 °C, flow at 10 L/min, nebulizer at 0 psig and capillary voltage at 3500 V. The sheath liquid used consisted of methanol: water (1:1, v/v) and two reference masses (20 μL of purine: 121.0509 and 20 μL of HP-0922: 922.0098) at a flow rate of 0.6 mL/min (1:100 of split ratio). The MS data in positive ionization were acquired using the Agilent MassHunter Workstation Software LC/MS Data Acquisition for 6200 series TOF/6500 series Q-TOF B 9.0.9044.0 (Agilent Technologies), and the raw data were inspected with the MassHunter Qualitative software (version B.08.00, Agilent Technologies) before data processing.

The serum sample analysis in negative ionization was performed using a 7100 capillary electrophoresis (Agilent Technologies) coupled to a 6224 time-of-flight mass spectrometer (TOF MS) (Agilent Technologies), equipped with an electrospray ionization (ESI) source. An Agilent Technologies fused polyvinyl alcohol capillary PVA (total length, 97.6 cm; internal diameter, 50 µm) was used for the separation of metabolites working in normal polarity. Before each analysis, the capillary was washed for 5 min (950 mbar) with background electrolyte (BGE) (0.1 M formic acid solution). Samples injection was performed during 50 s at 50 mbar and, to improve the analysis reproducibility, BGE was injected for 20 s at 100 mbar after each sample injection. The separation was performed with an internal pressure of 50 mbar at a voltage of -30 KV and at a constant temperature of 20 ° C, and the current observed under these conditions was 100 μA. The total analytical run time was 55 minutes. Mass spectrometry was operated in negative polarity, with a mass range 60–1000 m/z at a rate of 1.0 spectrum /s. Other parameters for the MS were: fragmentor at 125 V, skimmer at 65 V, OctopoleRFPeak at 750 V, drying gas temperature at 275 °C, flow at 10 L/min, nebulizer at 0 psig and capillary voltage at 2000 V. The sheath liquid used consisted of methanol: water (1:1, v/v) and two reference masses (20 μL of purine: 121.0509 and 20 μL of HP-0922: 922.0098) at a flow rate of 0.6 mL/min (1:100 of split ratio). The MS data in negative ionization were acquired using the Agilent MassHunter Workstation Software LC/MS Data Acquisition for 6200 series TOF/6500 series Q-TOF B 6.01.6172 SP1 (Agilent Technologies), and the raw data were inspected with the MassHunter Qualitative software (version B.08.00, Agilent Technologies) before data processing.


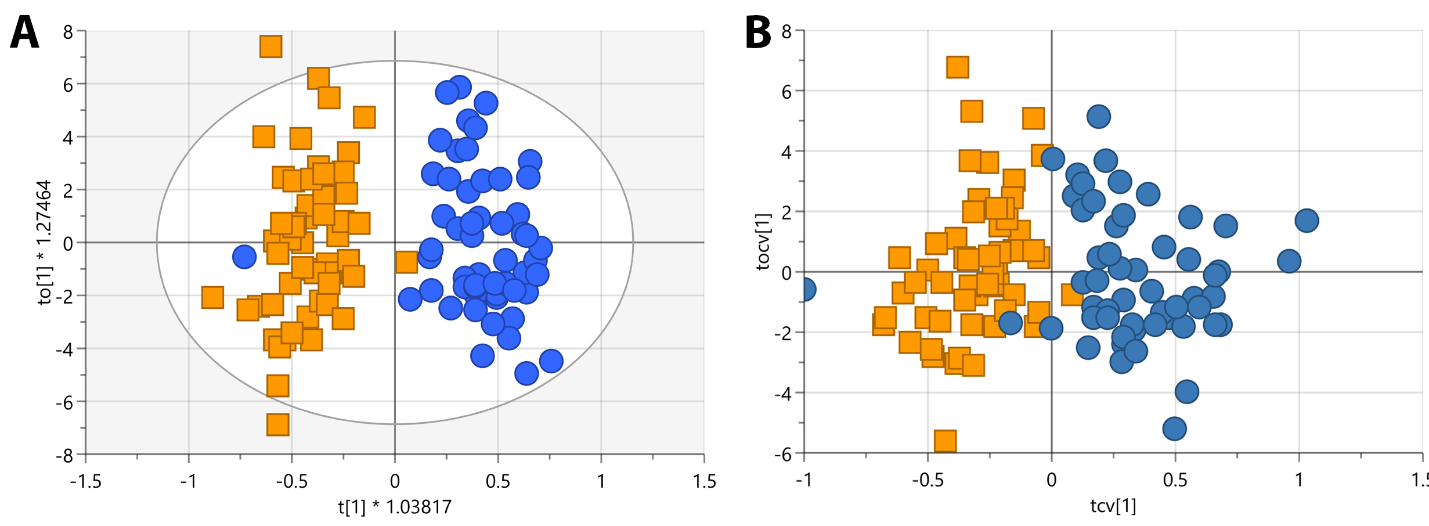


**Figure 1 Supplementary**. OPLS-DA LC-MS (+) score plot (blue dots, monogenic childhood obesity, orange square, idiopatic childhood obesity). A) OPLS-DA LC-MS (+) R^2^X= 0.72, R^2^Y= 0.82, Q^2^=0.64 with log10 transformation and Ctr scale. B) Scree plot of cross validation OPLS-DA LC-MS (+) model.


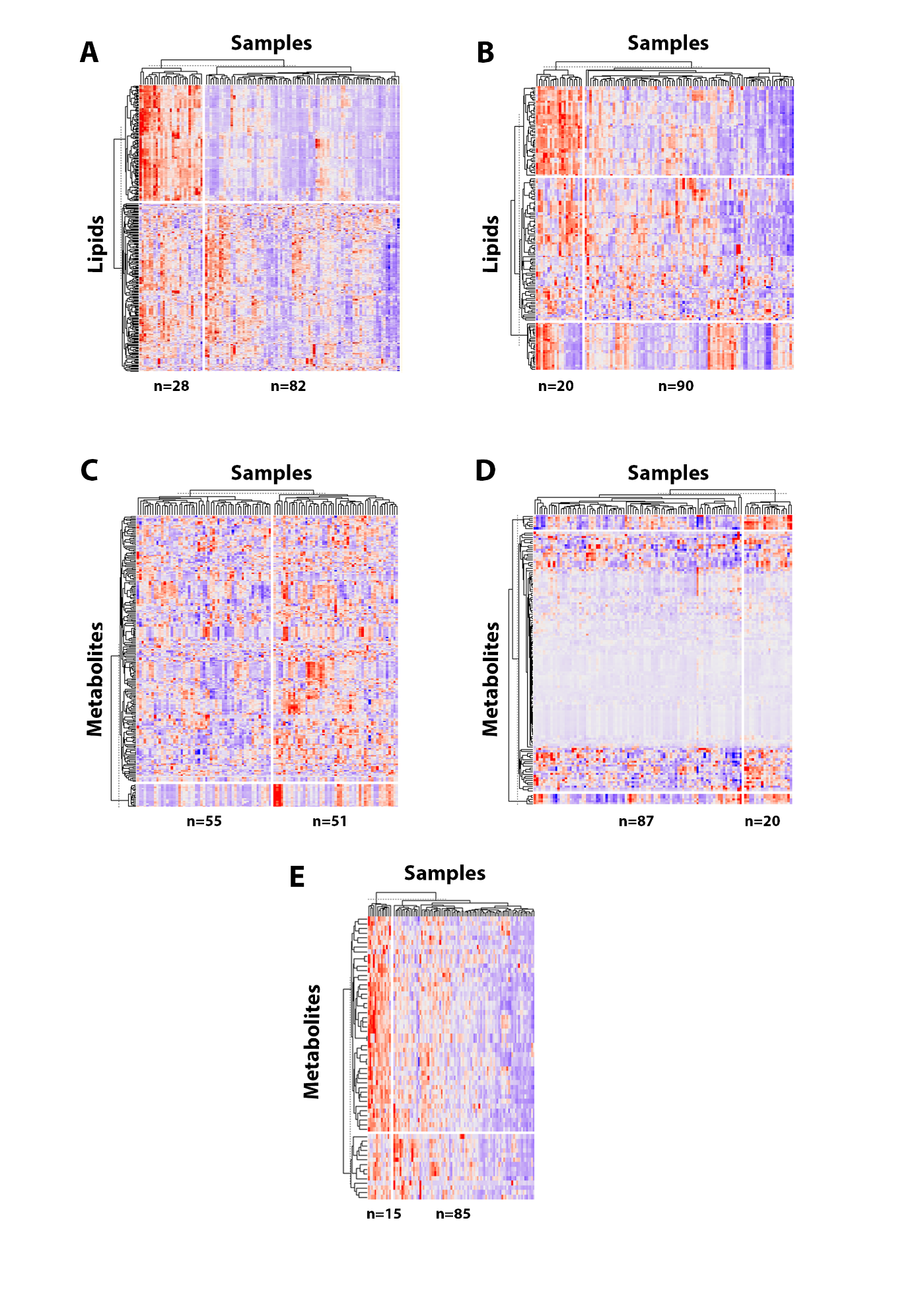


**Fig 2 Supplementary.** Hierarchical clustering of each of the platforms was carried out in R by applying a hierarchical algorithm with Euclidean distance and Ward method. The visualization has been performed using the R package "ComplexHeatmap” (Gu, Z. (2022) Complex Heatmap Visualization, iMeta. DOI: 10.1002/imt2.43.). **A)** LC-MS (+) **B)** LC-MS (-) **C)** CE-MS (+) **D)** CE-MS (-) **E)** GC-MS.

**Table 1 Supplementary**. Genetic variants present in each metabotype.

| HRSVs | **G1** | **G2** | **G3** |
| --- | --- | --- | --- |
| *POMC* | 4 | 0 | 0 |
| *SH2B1* | 5 | 0 | 0 |
| *CPE* | 2 | 0 | 2 |
| *PCSK1* | 4 | 1 | 0 |
| *NCOA1* | 6 | 0 | 1 |
| *MRAP2* | 4 | 0 | 0 |
| *MC4R* | 2 | 0 | 2 |
| *MC3R* | 4 | 1 | 0 |
| *SIM1* | 3 | 0 | 0 |
| *GRIK1* | 4 | 0 | 0 |
| *GRM7* | 1 | 1 | 1 |
| No variants | 35 | 7 | 10 |

**Table 2 Supplementary.**

| **Metabolite** | **Mass** | **RT** | **Analytical technique** | **Confidence Level** | **Adduct** | **Identification ions** | **GCMS derivative** |
| --- | --- | --- | --- | --- | --- | --- | --- |
| SM (d41:2) | 798.6603 | 11.91 | U(H)PLC-QTOF-MS/MS (+) | 3 | [M+H]^+^ | 184.0728; 799.6686; 781.6685; 104.1048 |  |
| SM (d42:3) | 810.6603 | 11.58 | U(H)PLC-QTOF-MS/MS (+) | 3 | [M+H]^+^ | 184.0727; 811.6648; 793.6524 |  |
| SM (d40:1) | 786.6612 | 12.06 | U(H)PLC-QTOF-MS/MS (+) | 3 | [M+H]^+^ | 184.0731; 787.6676 |  |
| SM (d39:1) | 772.6433 | 11.81 | U(H)PLC-QTOF-MS/MS (+) | 3 | [M+H]^+^ | 184.0730; 773.6514 |  |
| SM (d32:1) | 674.5355 | 5.89 | U(H)PLC-QTOF-MS/MS (+) | 3 | [M+H]^+^ | 184.0731; 675.5416 |  |
| SM (d36:2) | 728.5817 | 7.45 | U(H)PLC-QTOF-MS/MS (+) | 3 | [M+H]^+^ | 184.0726; 729.5888 |  |
| SM (d34:2) | 700.5503 | 6.1 | U(H)PLC-QTOF-MS/MS (+) | 3 | [M+H]^+^ | 184.0725; 701.5566 |  |
| SM (d34:0) | 704.5811 | 7.77 | U(H)PLC-QTOF-MS/MS (+) | 3 | [M+H]^+^ | 184.0721; 705.5918 |  |
| SM (d40:2) | 784.6449 | 11.57 | U(H)PLC-QTOF-MS/MS (+) | 3 | [M+H]^+^ | 184.0732; 785.6495 |  |
| SM (d38:1) | 758.6287 | 11.37 | U(H)PLC-QTOF-MS/MS (+) | 3 | [M+H]^+^ | 184.0724; 759.6352 |  |
| PC (16:0/16:0) | 733.5613 | 9.14 | U(H)PLC-QTOF-MS/MS (+) | 2 | [M+H]^+^ | 184.0733; 734.5706; 478.3358; 496.3398 |  |
| PC (O-34:1) | 745.5953 | 11.08 | U(H)PLC-QTOF-MS/MS (+) | 3 | [M+H]^+^ | 184.0722; 746.6056 |  |
| PC (O-40:4) | 823.649 | 12.06 | U(H)PLC-QTOF-MS/MS (+) | 3 | [M+H]^+^ | 184.0720; 824.6620 |  |
| PC (O-32:0) | 719.581 | 10.78 | U(H)PLC-QTOF-MS/MS (+) | 3 | [M+H]^+^ | 184.0723; 720.5883 |  |
| PC (O-38:4) | 795.6141 | 11.36 | U(H)PLC-QTOF-MS/MS (+) | 3 | [M+H]^+^ | 184.0718; 796.6227 |  |
| PC (O-36:5) | 765.5661 | 8.57 | U(H)PLC-QTOF-MS/MS (+) | 3 | [M+H]^+^ | 184.0725; 766.5729 |  |
| PC (16:0/18:2) | 757.5620 | 7.95 | U(H)PLC-QTOF-MS/MS (+) | 2 | [M+H]^+^ | 184.0727; 758.5688; 496.3468; 520.3347 |  |
| CE (18:2) | 648.5845 | 14.58 | U(H)PLC-QTOF-MS/MS (+) | 2 | [M+NH_4_]^+^ | 666.6090; 369.3523; 135.1134; 147.1173; 161.1334; 175.1450 |  |
| CE (20:4) | 672.5844 | 14.23 | U(H)PLC-QTOF-MS/MS (+) | 2 | [M+NH_4_]^+^ | 690.5182; 369.3508; 175.1481; 161.1325; 135.1142 |  |
| CE (18:1) | 650.6002 | 15.25 | U(H)PLC-QTOF-MS/MS (+) | 2 | [M+NH_4_]^+^ | 668.6175; 369.3510; 135.1160; 147.1168; 161.1325; 175.1481 |  |
| DG (36:4) | 616.5108 | 11.87 | U(H)PLC-QTOF-MS/MS (+) | 4 | [M+H]^+^ |  |  |
| TG (16:0_18:0_18:1) | 860.7833 | 14.95 | U(H)PLC-QTOF-MS/MS (+) | 3 | [M+NH_4_]^+^ | 878.8171; 861.7926; 605.5110; 579.5344; 577.5178 |  |
| TG (56:6) | 906.7676 | 13.74 | U(H)PLC-QTOF-MS/MS (+) | 3 | [M+NH_4_]^+^ | 924.8006 |  |
| TG (18:1_18:2_20:4) | 904.7520 | 13.55 | U(H)PLC-QTOF-MS/MS (+) | 3 | [M+NH_4_]^+^ | 922.7858; 625.5180; 623.5034; 601.5190 |  |
| TG (56:3) | 912.8146 | 14.93 | U(H)PLC-QTOF-MS/MS (+) | 3 | [M+NH_4_]^+^ | 930.8474 |  |
| TG (58:5) | 936.8146 | 13.26 | U(H)PLC-QTOF-MS/MS (+) | 4 | [M+NH_4_]^+^ |  |  |
| TG (56:2) | 914.8302 | 15.59 | U(H)PLC-QTOF-MS/MS (+) | 4 | [M+K]^+^ |  |  |
| TG (54:3) | 884.7833 | 14.36 | U(H)PLC-QTOF-MS/MS (+) | 3 | [M+NH_4_]^+^ | 902.8162 |  |
| TG (18:1_18:2_18:2) | 880.7520 | 13.55 | U(H)PLC-QTOF-MS/MS (+) | 3 | [M+NH_4_]^+^ | 898.7822; 601.5190; 599.5037 |  |
| TG (57:2) | 928.8459 | 14.11 | U(H)PLC-QTOF-MS/MS (+) | 4 | [M+NH_4_]^+^ |  |  |
| TG (53:3) | 870.7676 | 14.09 | U(H)PLC-QTOF-MS/MS (+) | 3 | [M+NH_4_]^+^ | 888.8018 |  |
| TG (54:4) | 882.7676 | 13.92 | U(H)PLC-QTOF-MS/MS (+) | 3 | [M+Na]^+^ | 905.7572 |  |
| TG (16:0_18:1_18:2) | 856.7520 | 13.92 | U(H)PLC-QTOF-MS/MS (+) | 3 | [M+NH_4_]^+^ | 874.7866; 601.5190; 577.5190; 575.5034 |  |
| Phenylalanine | 165.1891 | 13.545 | GC-q-MS | 2 | - | 120 | Phenylalanine, 1TMS derivative |
| Phenylalanine | 309.158 | 14.4173 | GC-q-MS | 2 | - | 218.1027 | Phenylalanine, 2TMS derivative |
| Oxalic acid | 234.074 | 7.923 | GC-q-MS | 2 | - | 190.1 | Oxalic acid, 2TMS derivative |
| Myo-Inositol | 612.301 | 19.2555 | GC-q-MS | 2 | - | 305.1432 | Myo-Inositol, 6TMS derivative |
| Cholesterol | 458.394 | 27.555 | GC-q-MS | 2 | - | 329 | Cholesterol, 1TMS derivative |
| Proline | 259.142 | 10.1572 | GC-q-MS | 2 | - | 142.1047 | Proline, 2TMS derivative |
| Serine | 105.0926 | 9.706 | GC-q-MS | 2 | - | 132 | Serine, 1TMS derivative |
| Serine | 321.161 | 11.0521 | GC-q-MS | 2 | - | 204.1234 | Serine, 3TMS derivative |
| Glycine | 219.111 | 7.6444 | GC-q-MS | 2 | - | 102.0734 | Glycine, 2TMS derivative |
| Glycine | 291.151 | 10.2794 | GC-q-MS | 2 | - | 174.1129 | Glycine, 3TMS derivative |
| Alanine | 233.127 | 7.415 | GC-q-MS | 2 | - | 116.089 | Alanine, 2TMS derivative |
| Methionine | 221.091 | 11.8076 | GC-q-MS | 2 | - | 104.053 | Methionine, TMS derivative |
| Methionine | 293.13 | 13.0542 | GC-q-MS | 2 | - | 176.0924 | Methionine, 2TMS derivative |
| 5-Oxoproline/ Pyroglutamic acid | 273.122 | 13.1458 | GC-q-MS | 2 | - | 230.1391 | 5-Oxoproline, 2TMS derivative |
| Valine | 117.1463 | 7.296 | GC-q-MS | 2 | - | 72 | Valine, 1TMS derivative |
| Valine | 261.158 | 8.9968 | GC-q-MS | 2 | - | 144.1203 | Valine, 2TMS derivative |
| Threonine | 335.177 | 11.3363 | GC-q-MS | 2 | - | 219.1105 | Threonine, 3TMS derivative |
| FA (20:4) | 304.2401 | 2.89 | U(H)PLC-QTOF-MS/MS (-) | 2 | [M-H]^-^ | 303.233 |  |
| FA (20:3) | 306.2557 | 3.3 | U(H)PLC-QTOF-MS/MS (-) | 2 | [M-H]^-^ | 305.2488 |  |
| FA (17:0) | 270.2559 | 3.83 | U(H)PLC-QTOF-MS/MS (-) | 2 | [M-H]^-^ | 269.2475 |  |
| FA (22:4) | 332.2716 | 3.59 | U(H)PLC-QTOF-MS/MS (-) | 2 | [M-H]^-^ | 331.2633 |  |
| FA (18:3) | 278.2245 | 2.47 | U(H)PLC-QTOF-MS/MS (-) | 2 | [M-H]^-^ | 277.217 |  |
| FA (18:0;O6) | 380.2395 | 3.57 | U(H)PLC-QTOF-MS/MS (-) | 4 | [M-H]^-^ |  |  |
| FA (14:0) | 228.2088 | 2.49 | U(H)PLC-QTOF-MS/MS (-) | 2 | [M-H]^-^ | 227.2014 |  |
| FA (22:5) | 330.2557 | 3.11 | U(H)PLC-QTOF-MS/MS (-) | 2 | [M-H]^-^ | 329.252 |  |
| FA (22:6) | 328.24 | 2.69 | U(H)PLC-QTOF-MS/MS (-) | 2 | [M-H]^-^ | 327.2351 |  |
| FA (14:1) | 226.1932 | 1.89 | U(H)PLC-QTOF-MS/MS (-) | 2 | [M-H]^-^ | 225.1875 |  |
| FAHFA (2:0_20:4) | 362.2434 | 2.99 | U(H)PLC-QTOF-MS/MS (-) | 4 | [M-H]^-^ |  |  |
| LPC (20:3/0:0) | 545.3476 | 2.47 | U(H)PLC-QTOF-MS/MS (+) | 2 | [M+H]^+^ | 184.0729; 546.3490; 528.3444 |  |
| PC (18:0_20:3) | 811.6082 | 10.86 | U(H)PLC-QTOF-MS/MS (+) | 3 | [M+H]^+^ | 184.0729; 812.6150; 524.3711 |  |
| PC (30:0) | 705.5324 | 7.29 | U(H)PLC-QTOF-MS/MS (+) | 3 | [M+H]^+^ | 184.0733; 706.5309 |  |
| PC (16:0_16:1) | 731.5452 | 7.53 | U(H)PLC-QTOF-MS/MS (+) | 3 | [M+H]^+^ | 184.0728; 732.5531; 496.3448 |  |
| PC (34:3) | 755.5452 | 7.14 | U(H)PLC-QTOF-MS/MS (+) | 3 | [M+H]^+^ | 184.0750; 756.5467 |  |
| PC (40:5) | 835.608 | 10.02 | U(H)PLC-QTOF-MS/MS (+) | 3 | [M+H]^+^ | 184.0720; 836.6150 |  |
| PC (33:1) | 745.5601 | 8.38 | U(H)PLC-QTOF-MS/MS (+) | 3 | [M+H]^+^ | 184.0733; 746.5685 |  |
| PC (18:0_18:1) | 787.6094 | 11.57 | U(H)PLC-QTOF-MS/MS (+) | 3 | [M+H]^+^ | 184.0727; 788.6150; 524.3684; |  |
| PC (18:0_22:4) | 837.6232 | 11.44 | U(H)PLC-QTOF-MS/MS (+) | 3 | [M+H]^+^ | 184.0735; 838.632; 524.3693 |  |
| PC (38:1) | 813.625 | 11.73 | U(H)PLC-QTOF-MS/MS (+) | 3 | [M+H]^+^ | 184.0717; 814.6299 |  |
| Glutamic acid | 147.0553 | 13.43 | CE-TOF-MS (+) | 2 | [M+H]^+^ | 148.0618; 84.0449; 102.0550; 130.0502 |  |
| Choline | 103.1009 | 9.64 | CE-TOF-MS (+) | 2 | [M+H]^+^ | 104.1075; 60.0816 |  |
| Aspartic acid | 133.0371 | 13.89 | CE-TOF-MS (+) | 2 | [M+H]^+^ | 134.0448; 88.0420; 116.0359 |  |
| Glutamine | 101.0487 | 13.27 | CE-TOF-MS (+) | 2 | [M+H]^+^ | 101.0720; 84.0436 | Fragment de Glutamine |
| Arginine | 115.0625 | 9.71 | CE-TOF-MS (+) | 2 | [M+H]^+^ | 116.0707; 70.0652 | Fragment de Arginine |

**REFERENCES**

1. Gonzalez-Riano C, Gradillas A, Barbas C. Exploiting the formation of adducts in mobile phases with ammonium fluoride for the enhancement of annotation in liquid chromatography-high resolution mass spectrometry based lipidomics. Journal of Chromatography Open. 2021 Nov;1:100018.
